# Risk and protective factors for relapse after residential substance use treatment: a systematic review and meta-analysis

**DOI:** 10.1186/s12888-026-08330-4

**Published:** 2026-07-23

**Authors:** Sajid Naeem, Amna Rasheed, Hasnain Ali

**Affiliations:** 1 School of Medical Science, Shandong Xiehe University, Jinan, 250109 P. R. China; 2 School of Basic Medical Science, Lanzhou University, Lanzhou, 730000 China; 3 National University of Medical Sciences, Rawalpindi, Pakistan

**Keywords:** Substance use disorder, Relapse prevention, Residential treatment, Self-efficacy, Psychological distress, Treatment duration, Aftercare, Risk factors, Protective factors

## Abstract

**Background:**

Relapse after residential treatment for substance use disorder (SUD) remains common, yet no prior systematic review and meta-analysis has comprehensively synthesized the risk and protective factors associated with post-discharge relapse. We aimed to identify and quantify factors associated with relapse after residential SUD treatment, while also examining whether outcomes differed by primary substance and by treatment type (mandatory versus voluntary).

**Methods:**

We conducted a PRISMA 2020 systematic review and meta-analysis registered with PROSPERO (CRD420251252324). PubMed, Embase, Scopus, PsycINFO, Web of Science, CINAHL Plus and grey literature were searched from inception to March 31, 2026. Primary studies of residential, inpatient, or therapeutic community SUD treatment reporting relapse-related outcomes were included. Random-effects inverse-variance models were used to pool risk ratios (RRs) for binary outcomes and standardized mean differences (SMDs) for continuous outcomes. Methodological quality was assessed using the NIH Quality Assessment Tool and RoB-1 for observational studies.

**Results:**

Sixty-one studies involving 18,693 participants were included: 60 cohort studies and one cross-sectional study. Twenty-seven studies were rated Good and 34 Fair. Interventions modestly increased abstinence (RR, 1.13; 95% CI, 1.03–1.25). Relapsed participants had higher depression scores (SMD, 1.45; 95% CI, 1.23–1.66), whereas psychological distress was lower among abstinent participants, and self-efficacy was higher among abstinent participants after harmonizing the direction of effects. Longer treatment duration was associated with better outcomes, although heterogeneity was extreme and the pooled magnitude should be interpreted cautiously. Peer-support-specific analysis showed improved abstinence, whereas broader continuing-care or intervention analyses produced a separate estimate. Alcohol was the dominant substance category, followed by polysubstance, opioids/heroin, cocaine, and methamphetamine use. Descriptive evidence suggested lower 6- to 12-month abstinence in heroin-focused than alcohol-focused cohorts, but no study compared substances within one framework. Two studies examined correctional or prison-based mandatory settings, and none compared mandatory with voluntary treatment.

**Conclusions:**

Relapse after residential SUD treatment was associated with greater depressive burden and lower self-efficacy, whereas peer support, longer treatment duration, and adherence-related interventions were associated with better outcomes. Evidence was insufficient to draw firm conclusions regarding substance-specific differences or mandatory versus voluntary treatment effects, underscoring the need for better-designed comparative studies that standardize relapse definitions, classify treatment voluntariness, and report outcomes by primary substance category.

**Clinical trial number:**

Not applicable.

**Supplementary Information:**

The online version contains supplementary material available at 10.1186/s12888-026-08330-4.

## Introduction

Relapse after residential treatment for substance use disorder (SUD) remains a major clinical and public health challenge, but its determinants are unlikely to be uniform across substance categories or treatment contexts [1]. Alcohol, opioids, stimulants, and polysubstance use differ in withdrawal burden, craving patterns, psychiatric comorbidity, overdose risk, and response to continuing care, all of which may influence post-discharge relapse trajectories [2, 3]. Likewise, the context in which residential treatment is delivered may shape outcomes, because voluntary treatment often reflects greater treatment readiness and engagement, whereas mandatory treatment may have differed in motivation, retention, and post-treatment recovery patterns [4]. Among the most persistent and complex public health issues worldwide, substance use disorders (SUDs) are implicated in approximately 11.8 million deaths per year, either directly or indirectly through associated health complications and accidents [1]. This wide variability in relapse outcomes highlights the importance of identifying which risk and protective factors are most consistently associated with post-discharge recovery. However, relapse rates, which seriously undermine long-term efficacy, remain high following available residential treatment programs designed for severe SUD cases [2]. Substantial evidence shows that more than 60% of individuals relapse within the first year after treatment, particularly during the critical transition period following discharge [3]. Among specific populations, such as U.S. veterans who are homeless, unemployed, or lack continued care, relapse rates can be as high as 94% [4]. Substance use and mental disorders remain major public-health concerns worldwide, with prevalence varying across countries, income levels, and sociodemographic groups [5].

The factors contributing to relapse are numerous and encompass biological, psychological, and social domains. Neurobiologically, activation of the nucleus accumbens by drug cues is a predictor of relapse risk, highlighting the persistent neuroadaptations resulting from chronic drug exposure [6]. Key psychosocial risk factors, such as impulsivity, poor self-efficacy, and negative emotional states, further amplify this vulnerability [7, 8]. Environmental factors, including unemployment, unstable housing, and limited social support, also significantly increase the risk of post-treatment relapse [9]. A growing body of research identifies protective factors that can mitigate relapse risk [10]. For instance, structured aftercare programs, supportive social networks, and participation in religious or spiritual activities significantly improve long-term abstinence outcomes [11]. Additionally, dispositional mindfulness and effective coping mechanisms serve as individual-level protective factors that build resilience and support sustained recovery [12, 13]. In this review, protective factors were conceptualized as modifiable personal resources and post-discharge supports that may buffer relapse vulnerability following residential treatment. These included individual-level resources such as self-efficacy, coping skills, motivation, and psychological resilience, as well as continuing-care components such as structured aftercare, peer support, medication adherence, and technology-assisted follow-up.

Although prior reviews have evaluated the effectiveness and characteristics of residential treatment services, quantitative syntheses that specifically integrate risk and protective factors for post-discharge relapse following residential SUD treatment remain limited, particularly across psychological and psychosocial domains in a single meta-analytic framework [14]. Existing studies have not adequately addressed two clinically important sources of heterogeneity: differences between substances and differences in treatment context. To date, no systematic review and meta-analysis has comprehensively synthesized risk and protective factors associated with relapse after residential SUD treatment while also examining whether relapse-related outcomes differ by primary substance and whether outcomes vary according to treatment type, particularly mandatory versus voluntary treatment exposure. Because residential SUD populations are heterogeneous, and because alcohol, opioids, stimulants, and polysubstance use may differ in relapse mechanisms, treatment response, psychiatric burden, and post-discharge recovery trajectories, a synthesis that explicitly examines substance-specific differences is needed. Treatment context may also influence recovery because mandatory and voluntary treatment pathways can differ in motivation, readiness to change, autonomy, therapeutic engagement, retention, legal supervision, and post-discharge support. Clarifying whether the existing literature can support substance-specific or treatment-context-specific conclusions is essential for clinicians, service planners, and policymakers seeking to tailor residential treatment and aftercare models to patient needs. Treatment context may have influenced recovery, as mandatory and voluntary treatment pathways can differ in motivation, engagement, retention, and relapse vulnerability. The review aimed to identify, quantify, and synthesize the key risk and protective factors associated with relapse following residential SUD treatment. A secondary aim was to examine whether available evidence supported hypotheses about differences between primary substance categories and the potential effect of mandatory versus voluntary treatment exposure. By integrating quantitative findings with descriptive evidence on substance type and treatment context, this review sought to inform clinical practice, treatment planning, and policy decisions aimed at improving long-term recovery outcomes after residential SUD treatment.

## Materials and methods

### Eligibility criteria

The systematic review and meta-analysis were conducted in accordance with the PRISMA 2020 statement [15]. The eligibility criteria were defined a priori in accordance with the PRISMA guidance and PROSPERO-registered protocol **(CRD420251252324)**. The review was designed to identify, quantify, and synthesize risk and protective factors associated with relapse after residential, inpatient, or therapeutic community treatment for substance use disorder (SUD). The review also prespecified a descriptive assessment of two clinically important sources of heterogeneity: primary substance category and treatment context, particularly mandatory versus voluntary treatment exposure. Eligible studies were required to include individuals who received residential, inpatient, or therapeutic community–type treatment for SUD and who had relapse-related outcomes measured after discharge or during follow-up. Relapse-related outcomes included return to substance use, abstinence, readmission, rehospitalization, documented clinical relapse, urine toxicology or breathalyzer-confirmed substance use, or composite recovery outcomes. Studies were included if they reported sufficient quantitative data to calculate or extract an effect estimate for at least one risk or protective factor.

### Information sources

A preliminary search was conducted to identify relevant keywords, followed by the development and implementation of a comprehensive search strategy across PubMed, Embase, Scopus, PsycINFO, Web of Science and CINAHL Plus, along with grey literature sources. The full search strategies for each database, including field tags, Boolean operators, and applied limits, are provided in Supplementary Appendix S1 to ensure reproducibility. The final search was conducted through March 31, 2026. In addition, reference lists of included studies and relevant reviews were manually screened to identify further eligible articles. The search strategy combined terms related to relapse outcomes (e.g., “relapse,” “recurrence,” “readmission,” “remission,” “abstinence,” “recovery”), residential treatment settings (e.g., “residential,” “therapeutic community,” “inpatient,” “detox,” “24-hour facility”), and substance use disorders (e.g., “substance,” “drug,” “alcohol,” “dependence,” “misuse,” “disorder”), using Boolean operators (AND/OR). All identified records were screened and assessed for eligibility based on the predefined inclusion and exclusion criteria.

### Study selection

All records identified through database and grey literature searches were imported into a reference-management file, and duplicates were removed before screening. Titles and abstracts were screened against the predefined eligibility criteria. Full texts were retrieved for all potentially eligible studies and assessed for final inclusion. Two reviewers, S.N. and A.R., independently screened titles, abstracts, and full-text articles. Disagreements were resolved through discussion, and unresolved disagreements were adjudicated by the senior author, H.A. Reasons for exclusion at the full-text stage were documented and summarized in the PRISMA flow diagram. The PRISMA flow diagram summarizes the identification, screening, eligibility, and inclusion process.

### Inclusion/Exclusion criteria

Eligibility criteria were defined using the PICO framework. We included primary studies of individuals who received residential/inpatient or therapeutic community-type SUD treatment that reported post-discharge relapse-related outcomes and examined factors associated with relapse or abstinence. Studies were eligible if they provided sufficient information to compute an effect size for at least one outcome of interest, including: (a) binary outcomes (e.g., relapse vs abstinence; readmission vs no readmission), enabling calculation of risk ratios (RRs) or odds ratios (ORs); and/or (b) continuous outcomes (e.g., depression severity, psychological distress, self-efficacy), enabling calculation of standardized mean differences (SMDs). We excluded non-primary research (reviews, editorials), non-English publications, and studies not conducted in residential treatment settings. Studies were not excluded on the basis of variation in primary substance type, treatment voluntariness, or relapse definition. These characteristics were extracted systematically and used to inform the descriptive synthesis, interpretation of subgroup patterns, and assessment of between-study heterogeneity. Treatment voluntariness was coded as unclear when it was not explicitly reported, and no assumptions were made regarding mandatory or voluntary treatment status.

### Operational definition of relapse across studies

Relapse was defined heterogeneously across primary studies. Definitions included: (1) self-reported return to substance use after discharge; (2) biological verification (e.g., urine toxicology, breathalyzer results); (3) health-system indicators such as treatment readmission, rehospitalization, or documented clinical relapse; and (4) composite definitions combining self-report with objective testing or administrative outcomes. When multiple relapse definitions were available within a study, we prioritized definitions that were (a) most objective (biological/clinical), and (b) most comparable across studies. We documented each study’s relapse operationalization and used this information in subgroup analysis by relapse definition type when sufficient studies were available.

### Review of methodological quality

Methodological quality was evaluated using RoB-1 and NIH Quality Assessment Tool for Observational Cohort and Cross-Sectional Studies (2021). This tool includes 14 evaluative questions and a grading system based on the number of “Yes” responses. Studies were rated as Good (10–14), Fair (5–9), or Poor (0–4).

### Data extraction

Data from eligible articles were extracted into predefined Excel spreadsheets. The extracted data included the first author’s name., year of publication, study design, study location, participant population, gender proportions, mean participant age, primary substance of use, duration of residential treatment, and follow-up duration after discharge. Data on risk or protective factors, odds ratios, and corresponding confidence intervals were also extracted for meta-analysis. Additional variables were extracted to address reviewer-requested subgroup concerns, including primary substance category and treatment voluntariness. Primary substance was coded as alcohol, opioid/heroin, cocaine/crack, methamphetamine/stimulant, polysubstance, petrol/inhalant, or mixed/unclear. Treatment context was coded as voluntary, mandatory/court-ordered, prison-based/correctional, justice-referred but not clearly mandatory, mixed, or unclear. Data extraction was performed independently by two reviewers, S.N. and A.R., to enhance accuracy and minimize bias. Inter-rater reliability for the extraction process was determined using Cohen’s kappa coefficient for key elements. Disagreements between reviewers were resolved through discussion and consensus, with arbitration by the senior author, H.A., when necessary. If disagreements persisted after discussion, the original article was re-evaluated, and the majority decision was entered into the final dataset.

### Effect size computation and harmonization

We grouped outcomes by measurement type. For binary outcomes (e.g., abstinence/relapse events), we pooled RRs when event counts and sample sizes were reported. For studies reporting odds ratios (ORs), we pooled log(OR) values and, where baseline risk information permitted, converted ORs to approximate RRs for comparability using established methods. For continuous outcomes (e.g., depression, psychological distress, self-efficacy), we computed standardized mean differences (SMDs; Hedges’ g) from post-treatment group means and standard deviations; when necessary, we derived SMDs from reported test statistics (*t*-values, F-values), confidence intervals, or standard errors using standard conversions. Directionality was harmonized so that negative SMD values consistently showed lower symptom burden or higher protective factor levels in the comparator group as defined in each analysis (e.g., abstinent vs relapsed).

### Statistical analysis

Statistical analyses were conducted using RStudio. Random-effects inverse-variance models were used for all pooled analyses because substantial clinical and methodological heterogeneity was expected across studies. Binary outcomes were summarized using RRs or odds ratios, as appropriate, with 95% confidence intervals. Continuous outcomes were summarized using standardized mean differences with 95% confidence intervals. Heterogeneity was quantified using I^2^ and τ^2^ statistics. I^2^ values greater than 50% were interpreted as evidence of substantial heterogeneity, although interpretation also considered the number of studies, overlap of confidence intervals, consistency of effect direction, clinical diversity, and outcome definitions. Statistical significance was evaluated using two-sided *p*-values, with *p* < 0.05 considered statistically significant. To explore sources of heterogeneity, we conducted prespecified subgroup analysis and, where feasible (≥10 studies per moderator), random-effects meta-regression examining moderators including: primary substance type, follow-up duration, relapse definition type (self-report vs biological vs administrative/composite), study design, and study quality rating. Robustness was assessed via leave-one-out sensitivity analysis and influence diagnostics. Meta-regression was considered only when at least 10 studies were available for a moderator, to avoid unstable estimates. Publication bias was assessed using funnel plots and by evaluating their symmetry. Funnel plots are most valid when more than 10 studies are included, with validity declining with fewer studies (Higgins and Thomas, 2023; Lin and Chu, 2017). When at least 10 studies were available for a pooled outcome, we additionally assessed small-study effects using Egger’s regression test; For outcomes with fewer than 10 studies, Egger’s test was not performed. We included risk/protective factors in pooled analyses when at least eight studies were available, considering this threshold sufficient to maintain statistical power and permit accurate analysis. Other potential methodological biases were assessed qualitatively.

### Quality appraisal and risk of bias assessment

Study quality and risk of bias were assessed according to study design using appropriate, design-specific tools. For observational studies (cohort and cross-sectional designs), we used the NIH Quality Assessment Tool for Observational Cohort and Cross-Sectional Studies, which evaluates methodological quality across 14 domains; studies were rated as Good, Fair, or Poor based on prespecified criteria. Risk of bias was assessed using the Cochrane Risk of Bias (RoB-1) tool. All assessments were conducted independently by two reviewers (H.A. and S.N.), with disagreements resolved through discussion or consultation with a senior author when necessary.

## Results

### Systematic literature search results

**Fig. 1:**
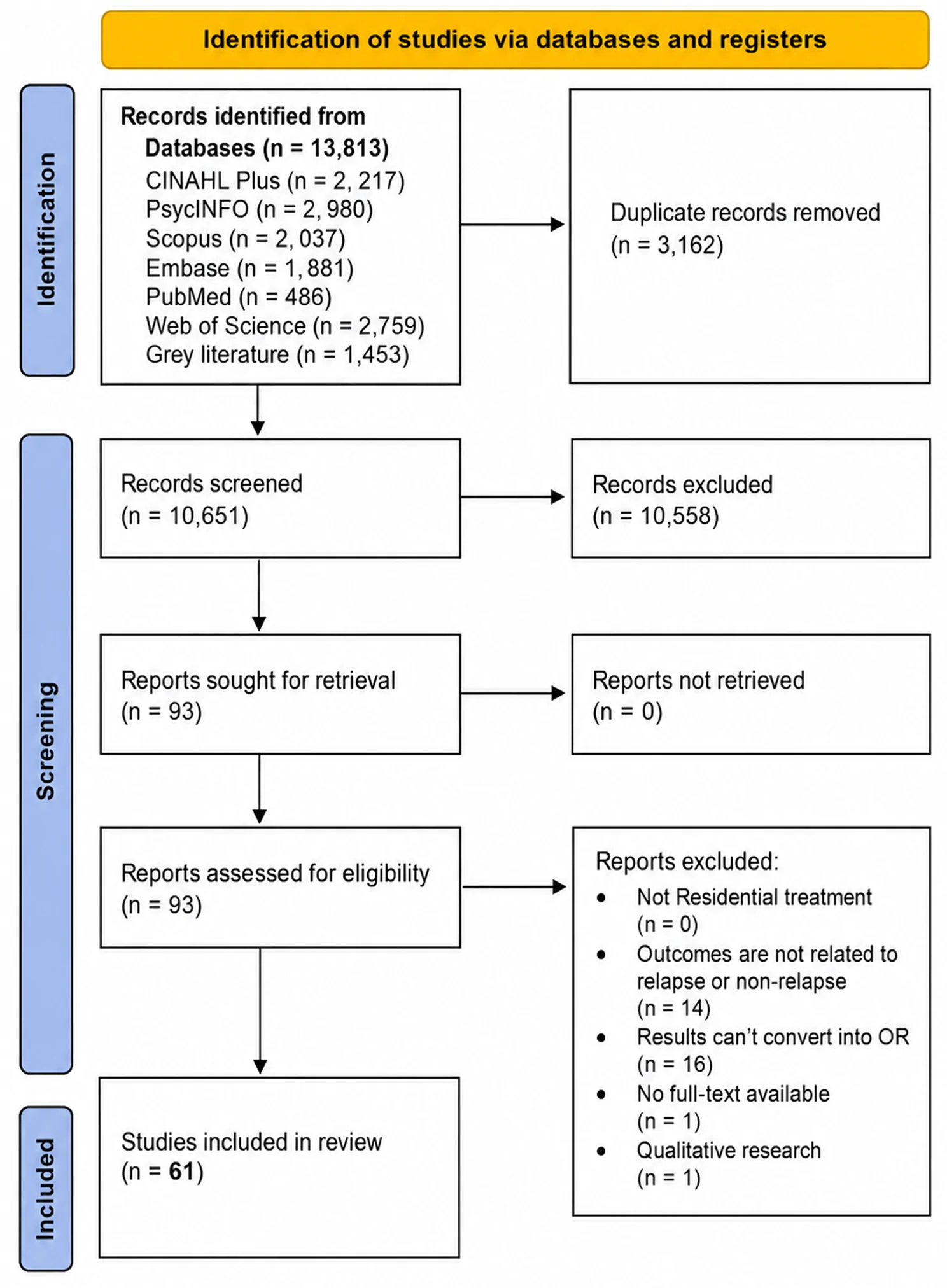
PRISMA flowchart showing the study selection process

Database and grey literature searches yielded 13,813 records. After removal of 3,162 duplicates, 10,651 unique records were screened by title and abstract, resulting in the exclusion of 10,558 articles primarily due to irrelevance. Ninety-three full-text articles were assessed for eligibility, and 61 studies met the inclusion criteria (Fig. 1). Study quality was evaluated using the NIH Quality Assessment Tool for Observational Cohort and Cross-Sectional Studies, with 27 studies rated Good and 34 rated Fair (Table 1, Appendix), indicating overall moderate-to-high methodological quality and a low-to-moderate risk of bias in the included evidence. Overall, 61 studies were included: 60 cohort studies, one cross-sectional study (Supplementary table 2). All included studies are fully referenced in the reference list and summarized with complete citations in Supplementary Table S2 [2–4, 6–10, 15–67]. The total sample comprised 18,693 participants. The mean age across studies was approximately 36.03 years [13, 14]. Alcohol was the most frequently studied primary substance (46 studies), followed by heroin, cocaine, and methamphetamine. Operational definitions of relapse varied across studies, including: (1) self-reported return to substance use after discharge; (2) biological verification (e.g., urine toxicology or breathalyzer results); (3) clinical or administrative indicators such as documented relapse, rehospitalization, or readmission to treatment; and (4) composite definitions combining more than one approach. The duration of residential treatment also varied substantially, ranging from a few weeks to over a year, contributing to clinical and methodological heterogeneity across studies.

**Table 1:** Quality assessment of included studies

Study	Item 1	Item 2	Item 3	Item 4	Item 5	Item 6	Item 7	Item 8	Item 9	Item 10	Item 11	Item 12	Item 13	Item 14	Overall quality
Anand et al. (2017)	Yes	Yes	Yes	Yes	Yes	Yes	NR	Yes	Yes	Yes	Yes	Yes	NR	Yes	Good
Andersson et al. (2021)	Yes	Yes	Yes	Yes	No	No	No	No	Yes	No	Yes	Yes	N/A	Yes	Fair
Barreno et al. (2018)	Yes	Yes	Yes	Yes	NR	No	No	No	Yes	Yes	Yes	N/A	CD	CD	Fair
Bauer et al. (2014)	Yes	Yes	Yes	Yes	Yes	Yes	NR	Yes	Yes	Yes	Yes	Yes	NR	Yes	Good
Bergman et al. (2014)	Yes	Yes	Yes	Yes	NR	No	No	No	Yes	Yes	Yes	N/A	CD	CD	Fair
Bergman et al. (2015)	Yes	Yes	Yes	Yes	Yes	No	No	Yes	Yes	Yes	Yes	Yes	N/A	Yes	Good
Best et al. (2020)	Yes	Yes	Yes	Yes	Yes	Yes	NR	Yes	Yes	Yes	Yes	Yes	NR	Yes	Good
Bosso et al. (2021)	Yes	CD	Yes	Yes	Yes	No	No	No	Yes	No	Yes	Yes	CD	Yes	Fair
Chong and Lopez (2008)	Yes	Yes	Yes	Yes	Yes	Yes	CD	CD	Yes	Yes	Yes	N/A	Yes	Yes	Good
Curran et al. (2000)	Yes	Yes	Yes	Yes	Yes	Yes	NR	Yes	Yes	Yes	Yes	Yes	NR	Yes	Good
Darke et al. (2006)	Yes	Yes	Yes	Yes	Yes	No	No	CD	Yes	Yes	Yes	NR	N/A	CD	Fair
Decker et al. (2017)	Yes	Yes	Yes	Yes	Yes	No	No	Yes	Yes	Yes	Yes	Yes	N/A	CD	Fair
Dingwall et al. (2012)	Yes	Yes	Yes	Yes	Yes	No	No	No	Yes	Yes	Yes	CD	N/A	Yes	Fair
Eslami et al. (2018)	Yes	Yes	CD	Yes	Yes	No	No	No	Yes	Yes	Yes	CD	N/A	Yes	Fair
Garcia-Rea & LePage (2010)	Yes	Yes	CD	Yes	Yes	Yes	CD	Yes	Yes	No	Yes	N/A	Yes	CD	Fair
Garland et al. (2012)	Yes	Yes	Yes	Yes	NR	No	No	No	Yes	Yes	Yes	N/A	CD	CD	Fair
Glass et al. (2017)	Yes	Yes	NR	Yes	Yes	Yes	CD	Yes	Yes	No	Yes	Yes	CD	Yes	Fair
Godley et al. (2006)	Yes	Yes	NR	Yes	No	Yes	CD	Yes	Yes	Yes	Yes	NR	Yes	Yes	Fair
Goldstein et al. (2001)	Yes	Yes	Yes	Yes	NR	No	No	Yes	Yes	No	Yes	Yes	N/A	Yes	Fair
Gossop et al. (1999)	Yes	Yes	Yes	Yes	No	No	No	No	Yes	Yes	Yes	Yes	N/A	Yes	Fair
Graser et al. (2022)	Yes	Yes	Yes	Yes	Yes	Yes	CD	CD	Yes	Yes	Yes	N/A	Yes	Yes	Good
Groh et al. (2007)	Yes	Yes	Yes	Yes	Yes	Yes	NR	Yes	Yes	Yes	Yes	Yes	NR	Yes	Good
Groh et al. (2011)	Yes	Yes	Yes	Yes	Yes	Yes	CD	CD	Yes	Yes	Yes	N/A	Yes	Yes	Good
Gustafson et al. (2014)	Yes	Yes	NR	Yes	No	Yes	CD	Yes	Yes	Yes	Yes	NR	Yes	Yes	Fair
Hajihosseini et al. (2019)	Yes	Yes	Yes	Yes	Yes	Yes	NR	Yes	Yes	Yes	Yes	Yes	NR	Yes	Good
Hser et al. (1999)	Yes	Yes	NR	Yes	Yes	No	No	No	Yes	No	Yes	NR	N/A	Yes	Fair
Jainchill et al. (2000)	Yes	Yes	CD	Yes	Yes	Yes	CD	Yes	Yes	No	Yes	N/A	Yes	CD	Fair
Januszko et al. (2021)	Yes	Yes	CD	Yes	Yes	Yes	CD	Yes	Yes	No	Yes	N/A	Yes	CD	Fair
Kabisa et al. (2021)	Yes	Yes	Yes	Yes	Yes	Yes	CD	CD	Yes	Yes	Yes	N/A	Yes	Yes	Good
Kelly et al. (2025)	Yes	Yes	Yes	Yes	No	No	No	No	Yes	No	Yes	Yes	N/A	Yes	Fair
Klein and Slaymaker (2011)	Yes	Yes	Yes	Yes	No	No	No	No	Yes	No	Yes	Yes	N/A	Yes	Fair
Klein et al. (2012)	Yes	Yes	Yes	Yes	Yes	Yes	NR	Yes	Yes	Yes	Yes	Yes	NR	Yes	Good
Kolla et al. (2015)	Yes	Yes	Yes	Yes	Yes	Yes	NR	Yes	Yes	Yes	Yes	Yes	NR	Yes	Good
Kornfield et al. (2018)	Yes	Yes	Yes	Yes	Yes	No	No	Yes	Yes	Yes	Yes	Yes	N/A	Yes	Good
Lang et al. (2024)	Yes	Yes	CD	Yes	No	Yes	NR	Yes	Yes	No	Yes	N/A	Yes	CD	Fair
Ludwig et al. (2013)	Yes	Yes	Yes	Yes	Yes	No	No	Yes	Yes	Yes	Yes	Yes	N/A	Yes	Good
Majer et al. (2018)	Yes	Yes	Yes	Yes	NR	No	No	No	Yes	Yes	Yes	N/A	CD	CD	Fair
Marcel et al. (2006)	Yes	Yes	NR	Yes	Yes	Yes	CD	Yes	Yes	No	Yes	Yes	CD	Yes	Fair
McKellar et al. (2009)	Yes	Yes	Yes	Yes	Yes	Yes	NR	Yes	Yes	Yes	Yes	Yes	NR	Yes	Good
McKetin et al. (2018)	Yes	Yes	Yes	Yes	Yes	No	No	Yes	Yes	Yes	Yes	Yes	N/A	Yes	Good
Mericle et al. (2019)	Yes	Yes	Yes	Yes	Yes	Yes	CD	CD	Yes	Yes	Yes	N/A	Yes	Yes	Good
Miller and Miller (2011)	Yes	Yes	NR	Yes	Yes	Yes	CD	Yes	Yes	No	Yes	Yes	CD	Yes	Fair
Mo and Deane (2016)	Yes	Yes	Yes	Yes	CD	Yes	NR	Yes	Yes	No	Yes	NR	Yes	Yes	Fair
M眉ller et al. (2019)	Yes	Yes	Yes	Yes	Yes	No	No	Yes	Yes	Yes	Yes	Yes	N/A	Yes	Good
Ober et al. (2023)	Yes	Yes	Yes	Yes	No	No	No	No	Yes	No	Yes	Yes	N/A	Yes	Fair
P. J. Kelly et al. (2023)	Yes	Yes	CD	Yes	No	Yes	NR	Yes	Yes	No	Yes	N/A	Yes	CD	Fair
Polcin and Henderson (2008)	Yes	Yes	NR	Yes	Yes	Yes	CD	Yes	Yes	No	Yes	Yes	CD	Yes	Fair
Polcin and Korcha (2017)	Yes	Yes	NR	Yes	No	Yes	CD	Yes	Yes	Yes	Yes	NR	Yes	Yes	Fair
Polcin et al. (2010)	Yes	Yes	Yes	Yes	Yes	No	No	No	Yes	No	Yes	Yes	N/A	NR	Fair
Ponzer et al. (2002)	Yes	Yes	Yes	Yes	No	No	No	No	Yes	No	Yes	Yes	N/A	Yes	Fair
Proctor et al. (2017)	Yes	CD	Yes	Yes	Yes	No	NR	Yes	Yes	Yes	Yes	Yes	N/A	CD	Fair
Rifai et al. (2006)	Yes	Yes	Yes	Yes	No	Yes	NR	Yes	Yes	No	Yes	NR	Yes	Yes	Fair
Rollins et al. (2005)	Yes	Yes	Yes	Yes	Yes	Yes	NR	Yes	Yes	Yes	Yes	Yes	NR	Yes	Good
Sannibale et al. (2003)	Yes	Yes	CD	Yes	Yes	Yes	CD	Yes	Yes	No	Yes	N/A	Yes	CD	Fair
Saur et al. (2022)	Yes	Yes	Yes	Yes	No	Yes	NR	Yes	Yes	No	Yes	NR	Yes	Yes	Fair
Savage and McCabe (1973)	Yes	Yes	Yes	Yes	Yes	No	No	Yes	Yes	No	Yes	Yes	N/A	CD	Fair
Sinclair et al. (2021)	Yes	Yes	Yes	Yes	Yes	Yes	CD	CD	Yes	Yes	Yes	N/A	Yes	Yes	Good
Suter et al. (2011)	Yes	Yes	NR	Yes	Yes	No	No	No	Yes	No	Yes	NR	N/A	Yes	Fair
van Hagen et al. (2019)	Yes	Yes	NR	Yes	No	Yes	CD	Yes	Yes	Yes	Yes	NR	Yes	Yes	Fair
Vanderplasschen et al. (2009)	Yes	Yes	Yes	Yes	Yes	No	No	Yes	Yes	Yes	Yes	Yes	N/A	Yes	Good
Welsh (2007)	Yes	Yes	Yes	Yes	Yes	No	No	No	Yes	Yes	Yes	CD	N/A	CD	Fair
Study	Item 1	Item 2	Item 3	Item 4	Item 5	Item 6	Item 7	Item 8	Item 9	Item 10	Item 11	Item 12	Item 13	Item 14	Overall quality
Anand et al. (2017)	Yes	Yes	Yes	Yes	Yes	Yes	NR	Yes	Yes	Yes	Yes	Yes	NR	Yes	Good
Andersson et al. (2021)	Yes	Yes	Yes	Yes	No	No	No	No	Yes	No	Yes	Yes	N/A	Yes	Fair
Barreno et al. (2018)	Yes	Yes	Yes	Yes	NR	No	No	No	Yes	Yes	Yes	N/A	CD	CD	Fair
Bauer et al. (2014)	Yes	Yes	Yes	Yes	Yes	Yes	NR	Yes	Yes	Yes	Yes	Yes	NR	Yes	Good
Bergman et al. (2014)	Yes	Yes	Yes	Yes	NR	No	No	No	Yes	Yes	Yes	N/A	CD	CD	Fair
Bergman et al. (2015)	Yes	Yes	Yes	Yes	Yes	No	No	Yes	Yes	Yes	Yes	Yes	N/A	Yes	Good
Best et al. (2020)	Yes	Yes	Yes	Yes	Yes	Yes	NR	Yes	Yes	Yes	Yes	Yes	NR	Yes	Good
Bosso et al. (2021)	Yes	CD	Yes	Yes	Yes	No	No	No	Yes	No	Yes	Yes	CD	Yes	Fair
Chong and Lopez (2008)	Yes	Yes	Yes	Yes	Yes	Yes	CD	CD	Yes	Yes	Yes	N/A	Yes	Yes	Good
Curran et al. (2000)	Yes	Yes	Yes	Yes	Yes	Yes	NR	Yes	Yes	Yes	Yes	Yes	NR	Yes	Good
Darke et al. (2006)	Yes	Yes	Yes	Yes	Yes	No	No	CD	Yes	Yes	Yes	NR	N/A	CD	Fair
Decker et al. (2017)	Yes	Yes	Yes	Yes	Yes	No	No	Yes	Yes	Yes	Yes	Yes	N/A	CD	Fair
Dingwall et al. (2012)	Yes	Yes	Yes	Yes	Yes	No	No	No	Yes	Yes	Yes	CD	N/A	Yes	Fair
Eslami et al. (2018)	Yes	Yes	CD	Yes	Yes	No	No	No	Yes	Yes	Yes	CD	N/A	Yes	Fair
Garcia-Rea & LePage (2010)	Yes	Yes	CD	Yes	Yes	Yes	CD	Yes	Yes	No	Yes	N/A	Yes	CD	Fair
Garland et al. (2012)	Yes	Yes	Yes	Yes	NR	No	No	No	Yes	Yes	Yes	N/A	CD	CD	Fair
Glass et al. (2017)	Yes	Yes	NR	Yes	Yes	Yes	CD	Yes	Yes	No	Yes	Yes	CD	Yes	Fair
Godley et al. (2006)	Yes	Yes	NR	Yes	No	Yes	CD	Yes	Yes	Yes	Yes	NR	Yes	Yes	Fair
Goldstein et al. (2001)	Yes	Yes	Yes	Yes	NR	No	No	Yes	Yes	No	Yes	Yes	N/A	Yes	Fair
Gossop et al. (1999)	Yes	Yes	Yes	Yes	No	No	No	No	Yes	Yes	Yes	Yes	N/A	Yes	Fair
Graser et al. (2022)	Yes	Yes	Yes	Yes	Yes	Yes	CD	CD	Yes	Yes	Yes	N/A	Yes	Yes	Good
Groh et al. (2007)	Yes	Yes	Yes	Yes	Yes	Yes	NR	Yes	Yes	Yes	Yes	Yes	NR	Yes	Good
Groh et al. (2011)	Yes	Yes	Yes	Yes	Yes	Yes	CD	CD	Yes	Yes	Yes	N/A	Yes	Yes	Good
Gustafson et al. (2014)	Yes	Yes	NR	Yes	No	Yes	CD	Yes	Yes	Yes	Yes	NR	Yes	Yes	Fair
Hajihosseini et al. (2019)	Yes	Yes	Yes	Yes	Yes	Yes	NR	Yes	Yes	Yes	Yes	Yes	NR	Yes	Good
Hser et al. (1999)	Yes	Yes	NR	Yes	Yes	No	No	No	Yes	No	Yes	NR	N/A	Yes	Fair
Jainchill et al. (2000)	Yes	Yes	CD	Yes	Yes	Yes	CD	Yes	Yes	No	Yes	N/A	Yes	CD	Fair
Januszko et al. (2021)	Yes	Yes	CD	Yes	Yes	Yes	CD	Yes	Yes	No	Yes	N/A	Yes	CD	Fair
Kabisa et al. (2021)	Yes	Yes	Yes	Yes	Yes	Yes	CD	CD	Yes	Yes	Yes	N/A	Yes	Yes	Good
Kelly et al. (2025)	Yes	Yes	Yes	Yes	No	No	No	No	Yes	No	Yes	Yes	N/A	Yes	Fair
Klein and Slaymaker (2011)	Yes	Yes	Yes	Yes	No	No	No	No	Yes	No	Yes	Yes	N/A	Yes	Fair
Klein et al. (2012)	Yes	Yes	Yes	Yes	Yes	Yes	NR	Yes	Yes	Yes	Yes	Yes	NR	Yes	Good
Kolla et al. (2015)	Yes	Yes	Yes	Yes	Yes	Yes	NR	Yes	Yes	Yes	Yes	Yes	NR	Yes	Good
Kornfield et al. (2018)	Yes	Yes	Yes	Yes	Yes	No	No	Yes	Yes	Yes	Yes	Yes	N/A	Yes	Good
Lang et al. (2024)	Yes	Yes	CD	Yes	No	Yes	NR	Yes	Yes	No	Yes	N/A	Yes	CD	Fair
Ludwig et al. (2013)	Yes	Yes	Yes	Yes	Yes	No	No	Yes	Yes	Yes	Yes	Yes	N/A	Yes	Good
Majer et al. (2018)	Yes	Yes	Yes	Yes	NR	No	No	No	Yes	Yes	Yes	N/A	CD	CD	Fair
Marcel et al. (2006)	Yes	Yes	NR	Yes	Yes	Yes	CD	Yes	Yes	No	Yes	Yes	CD	Yes	Fair
McKellar et al. (2009)	Yes	Yes	Yes	Yes	Yes	Yes	NR	Yes	Yes	Yes	Yes	Yes	NR	Yes	Good
McKetin et al. (2018)	Yes	Yes	Yes	Yes	Yes	No	No	Yes	Yes	Yes	Yes	Yes	N/A	Yes	Good
Mericle et al. (2019)	Yes	Yes	Yes	Yes	Yes	Yes	CD	CD	Yes	Yes	Yes	N/A	Yes	Yes	Good
Miller and Miller (2011)	Yes	Yes	NR	Yes	Yes	Yes	CD	Yes	Yes	No	Yes	Yes	CD	Yes	Fair
Mo and Deane (2016)	Yes	Yes	Yes	Yes	CD	Yes	NR	Yes	Yes	No	Yes	NR	Yes	Yes	Fair
M眉ller et al. (2019)	Yes	Yes	Yes	Yes	Yes	No	No	Yes	Yes	Yes	Yes	Yes	N/A	Yes	Good
Ober et al. (2023)	Yes	Yes	Yes	Yes	No	No	No	No	Yes	No	Yes	Yes	N/A	Yes	Fair
P. J. Kelly et al. (2023)	Yes	Yes	CD	Yes	No	Yes	NR	Yes	Yes	No	Yes	N/A	Yes	CD	Fair
Polcin and Henderson (2008)	Yes	Yes	NR	Yes	Yes	Yes	CD	Yes	Yes	No	Yes	Yes	CD	Yes	Fair
Polcin and Korcha (2017)	Yes	Yes	NR	Yes	No	Yes	CD	Yes	Yes	Yes	Yes	NR	Yes	Yes	Fair
Polcin et al. (2010)	Yes	Yes	Yes	Yes	Yes	No	No	No	Yes	No	Yes	Yes	N/A	NR	Fair
Ponzer et al. (2002)	Yes	Yes	Yes	Yes	No	No	No	No	Yes	No	Yes	Yes	N/A	Yes	Fair
Proctor et al. (2017)	Yes	CD	Yes	Yes	Yes	No	NR	Yes	Yes	Yes	Yes	Yes	N/A	CD	Fair
Rifai et al. (2006)	Yes	Yes	Yes	Yes	No	Yes	NR	Yes	Yes	No	Yes	NR	Yes	Yes	Fair
Rollins et al. (2005)	Yes	Yes	Yes	Yes	Yes	Yes	NR	Yes	Yes	Yes	Yes	Yes	NR	Yes	Good
Sannibale et al. (2003)	Yes	Yes	CD	Yes	Yes	Yes	CD	Yes	Yes	No	Yes	N/A	Yes	CD	Fair
Saur et al. (2022)	Yes	Yes	Yes	Yes	No	Yes	NR	Yes	Yes	No	Yes	NR	Yes	Yes	Fair
Savage and McCabe (1973)	Yes	Yes	Yes	Yes	Yes	No	No	Yes	Yes	No	Yes	Yes	N/A	CD	Fair
Sinclair et al. (2021)	Yes	Yes	Yes	Yes	Yes	Yes	CD	CD	Yes	Yes	Yes	N/A	Yes	Yes	Good
Suter et al. (2011)	Yes	Yes	NR	Yes	Yes	No	No	No	Yes	No	Yes	NR	N/A	Yes	Fair
van Hagen et al. (2019)	Yes	Yes	NR	Yes	No	Yes	CD	Yes	Yes	Yes	Yes	NR	Yes	Yes	Fair
Vanderplasschen et al. (2009)	Yes	Yes	Yes	Yes	Yes	No	No	Yes	Yes	Yes	Yes	Yes	N/A	Yes	Good
Welsh (2007)	Yes	Yes	Yes	Yes	Yes	No	No	No	Yes	Yes	Yes	CD	N/A	CD	Fair

## Meta-analysis results

### Outcomes of relapse prevention interventions

**Fig. 2:**
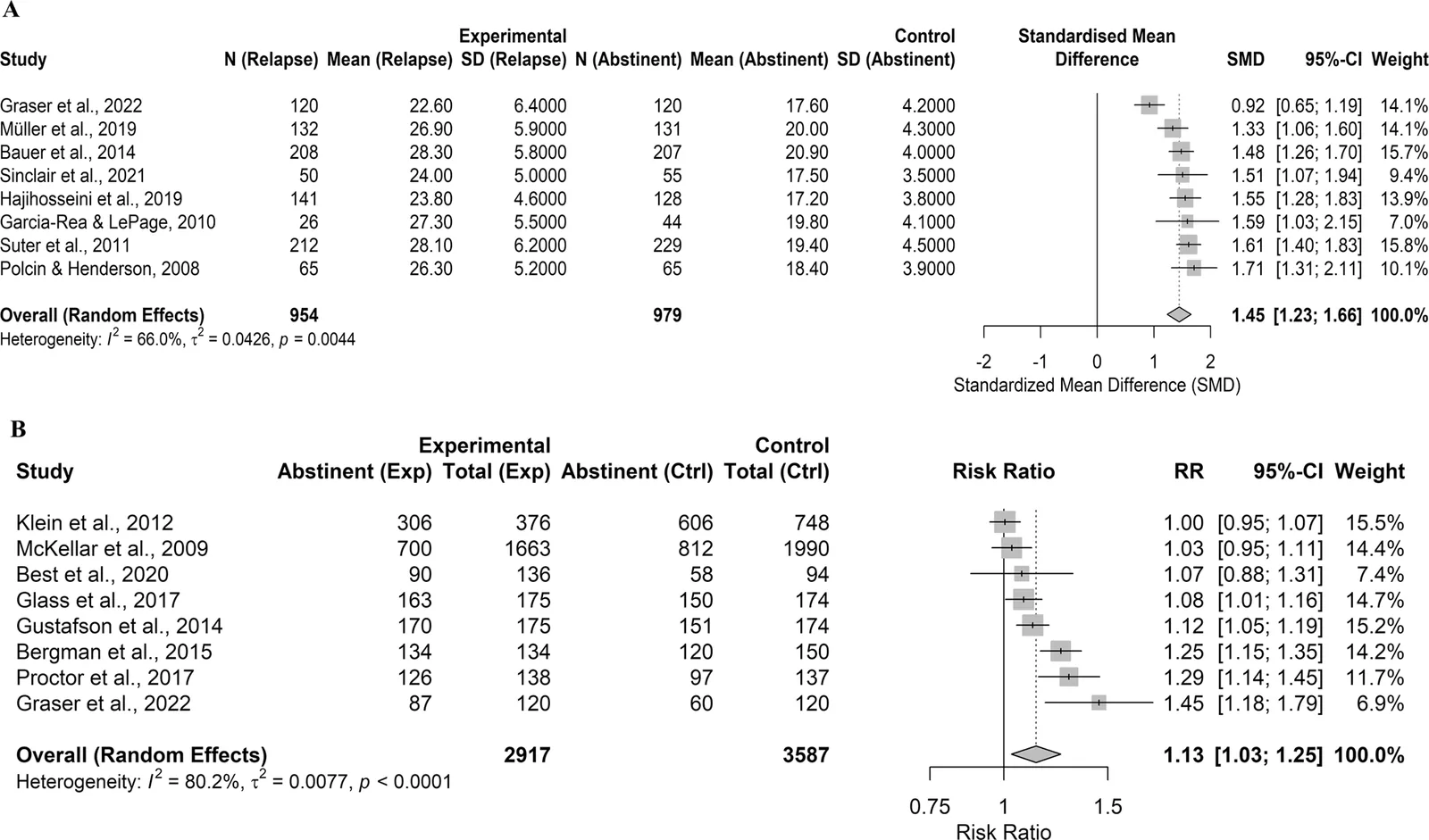
Forest plot comparing depression scores between relapsed and abstinent participants after residential substance use treatment, showing significantly higher depression scores among relapsed participants (SMD = 1.45; 95% CI: 1.23–1.66; I^2^ = 66.0%). **B.** Forest plot showing abstinence outcomes with continuing-care or relapse-prevention interventions, indicating a modest improvement in abstinence compared with control conditions (RR = 1.13; 95% CI: 1.03–1.25; I^2^ = 80.2%)

The meta-analysis evaluated the impact of different interventions on the rates of abstinence and psychological measures, particularly the severity of depression, among individuals in residential treatment for substance use. Eight studies included in the pooled analysis reported the RRs of abstinence between the experimental groups and the control groups, as shown in Fig. 2A. The analysis included 2,917 participants in the intervention groups and 3,587 in the control groups. The individual study RRs ranged from 1.00 [0.95, 1.07] for Klein et al. (2012) to 1.45 [1.18, 1.79] for Graser et al., with the overall random-effects model yielding RR = 1.13 [1.03, 1.25], implying that the interventions produced a modest but statistically significant improvement in abstinence. Heterogeneity was high (I^2^ = 80.2%, *p* < 0.0001) suggesting variability between studies (Fig. 2A). Figure 2B shows a forest plot for the standardized mean difference (SMD) between relapsed and abstinent subjects in depression scores. The final analysis included 954 relapse cases and 979 abstinent individuals. Participants who relapsed had higher depression scores, ranging from an SMD of 0.92 [0.65, 1.19] to 1.71 [1.31, 2.11]. The final estimation of SMD was 1.45 [1.23, 1.66], meaning that relapse is significantly associated with a higher depressive symptom burden. The heterogeneity level was moderate (I^2^ = 66.0%, *p* = 0.0044) (Fig. 2B).

### Psychological distress, treatment duration, and gender differences in relapse

**Fig. 3:**
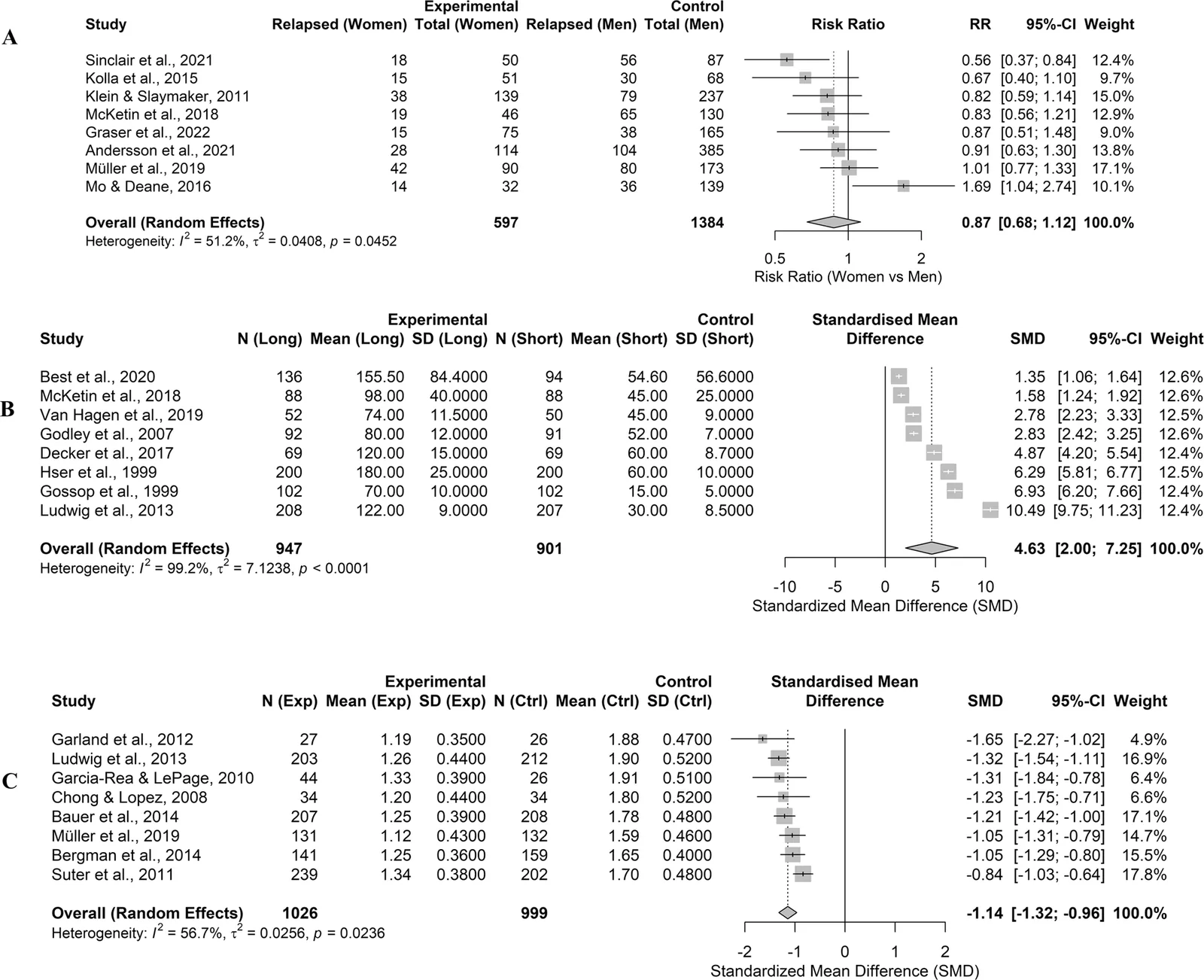
(**A**) Forest plot comparing relapse risk in women versus men after residential substance use treatment (RR = 0.87; 95% CI: 0.68–1.12). (**B**) Forest plot comparing longer-duration versus shorter-duration residential treatment, showing more favorable post-treatment outcomes with longer treatment duration (SMD = 4.63; 95% CI: 2.00–7.25). (**C**) Forest plot comparing psychological distress scores, showing lower psychological distress in abstinent or intervention groups than in comparison groups (SMD = −1.14; 95% CI: −1.32 to −0.96)

These meta-analyses focused on three major areas related to treatment outcomes for substance use: psychological distress, duration of treatment, and gender-relapse differences. Each area provides a different perspective on relapse prevention strategies and enhancing long-term recovery. The forest plot shown in (3A) summarizes the SMD of distress scores between the experimental and control groups across eight studies. In total, there were 1026 experimental and 999 control subjects. All studies showed a substantial reduction in distress for the experimental subjects. SMD ranged from −0.84 [−1.03, −0.64] for Suter et al. (2011) to −1.65 [−2.27, −1.02] for Garland et al. (2012), with an overall effect of −1.14 [−1.32, −0.96], favoring the experimental interventions [68, 69]. The heterogeneity was moderate (I^2^ = 56.7%, *p* = 0.0236), suggesting effects were directionally consistent but varied in magnitude (Fig. 3A).

Figure 3B compares outcomes between participants who received longer-duration versus shorter-duration residential treatment across eight studies. Residential treatment completion among women may also depend on family-centred support, parenting needs, and treatment engagement [70]. Pregnant and postpartum women may experience distinct residential-treatment outcomes according to treatment period and service context [71]. In total, 947 participants were included in the long-duration group and 901 in the short-duration group. Overall, outcomes were higher among individuals receiving longer treatment, yielding a very large pooled standardized effect (SMD = 4.63; 95% CI: 2.00 to 7.25). The largest individual effect was reported by Ludwig et al. (2013) (SMD = 10.49; 95% CI: 9.75 to 11.23). However, heterogeneity was extreme (I^2^ = 99.2%, *p* < 0.0001), indicating substantial between-study variability. Because “treatment duration” and outcome constructs were operationalized inconsistently across studies (e.g., different cut-offs defining short vs long programs and non-uniform outcome measures), this pooled SMD should be interpreted with caution (Fig. 3B). Figure 3Cexamines the RR used to analyze sex differences regarding relapse rates. Taking eight studies, encompassing 597 women and 1384 men, the overall RR was 0.87 [0.68, 1.12], meaning relapse rates in women were lower than those among men. This difference was not statistically significant. Only one study (Sinclair et al., 2021) reported a lower relapse risk in females (RR = 0.56 [0.37, 0.84]), but Mo and Deane (2016) reported a higher relapse in females (RR = 1.69 [1.04, 2.74]). Heterogeneity was moderate (I^2^ = 51.2%, *p* = 0.0452), suggesting some variability among the populations studied or the definitions of relapse employed (Fig. 3C).

### Impact of peer support and digital interventions on abstinence outcomes: meta-analytic findings

**Fig. 4:**
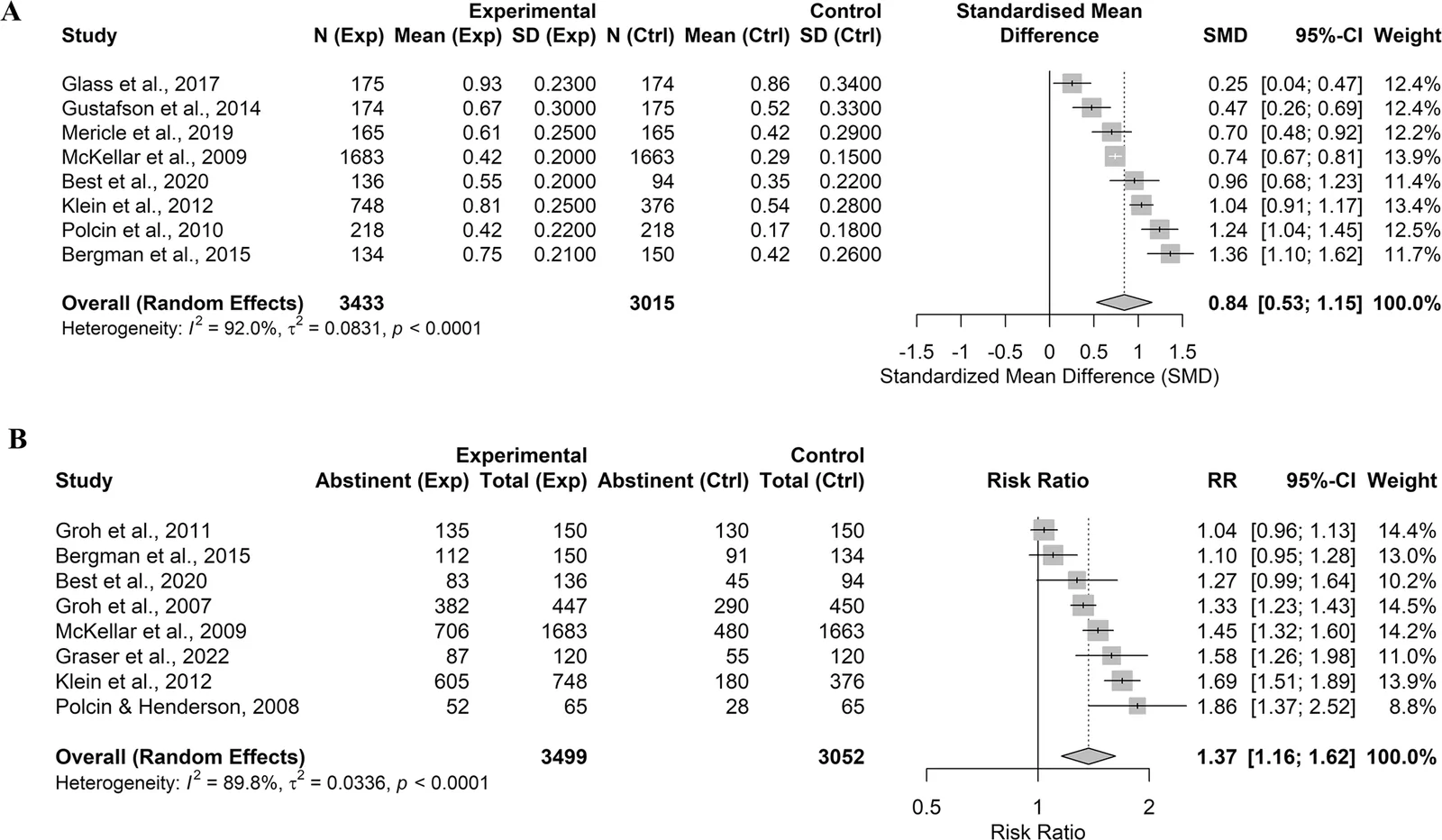
(**A**) Forest plot showing continuous recovery-related outcomes in intervention versus control groups, favoring the intervention group (SMD = 0.84; 95% CI: 0.53–1.15). (**B**) Forest plot showing abstinence outcomes in intervention versus control groups, indicating a higher likelihood of abstinence with intervention or recovery-support approaches (RR = 1.37; 95% CI: 1.16–1.62)

The analysis in Fig. 4 Ashowed that the intervention produced a consistent improvement in continuous outcomes when compared with control groups. Standardized mean differences were extracted from each study. Effect sizes ranged from 0.25 (Glass et al., 2017) to 1.36 (Bergman et al., 2015). Several trials reported moderate benefits, including Gustafson et al. (2014) with an SMD of 0.47, Mericle et al. (2019) with 0.70, and McKellar et al. (2009) with 0.74. Stronger effects were reported by Best et al. (2020) with 0.96, Polcin et al. (2010) with 1.24, and Klein et al. (2012) with 1.04. Across 3433 participants in the intervention arm and 3015 in the control arm, the pooled standardized mean difference was 0.84 [95% CI: 0.53–1.15]. Heterogeneity was high (I^2^ = 92.0%, *p* < 0.0001), suggesting substantial variation in effect magnitude among studies. Overall, the intervention consistently improved continuous treatment-related outcomes compared with standard care (Fig. 4A).

The analysis showed abstinence outcomes using RR. Individual study effects ranged from minimal benefit to large increases in abstinence. Groh et al. (2011) reported an RR of 1.04, Bergman et al. (2015) reported 1.10, and Best et al. (2020) reported 1.27. Moderate to strong effects were noted in multiple studies, including Groh et al. (2007) with 1.33, McKellar et al. (2009) with 1.45, Graser et al. (2022) with 1.58, Klein et al. (2012) with 1.69, and Polcin and Henderson (2008) with 1.86. The pooled sample included 3499 individuals in the intervention group and 3052 in the control group. The combined RR was 1.37 [95% CI: 1.16–1.62], indicating a higher likelihood of abstinence in the intervention group. Heterogeneity remained high (I^2^ = 89.8%, *p* < 0.0001). The intervention increased the probability of abstinence when compared with control conditions (Fig. 4B).

### Comprehensive analysis of peer support, social support, self-efficacy, and mental health factors on abstinence and relapse

**Fig. 5:**
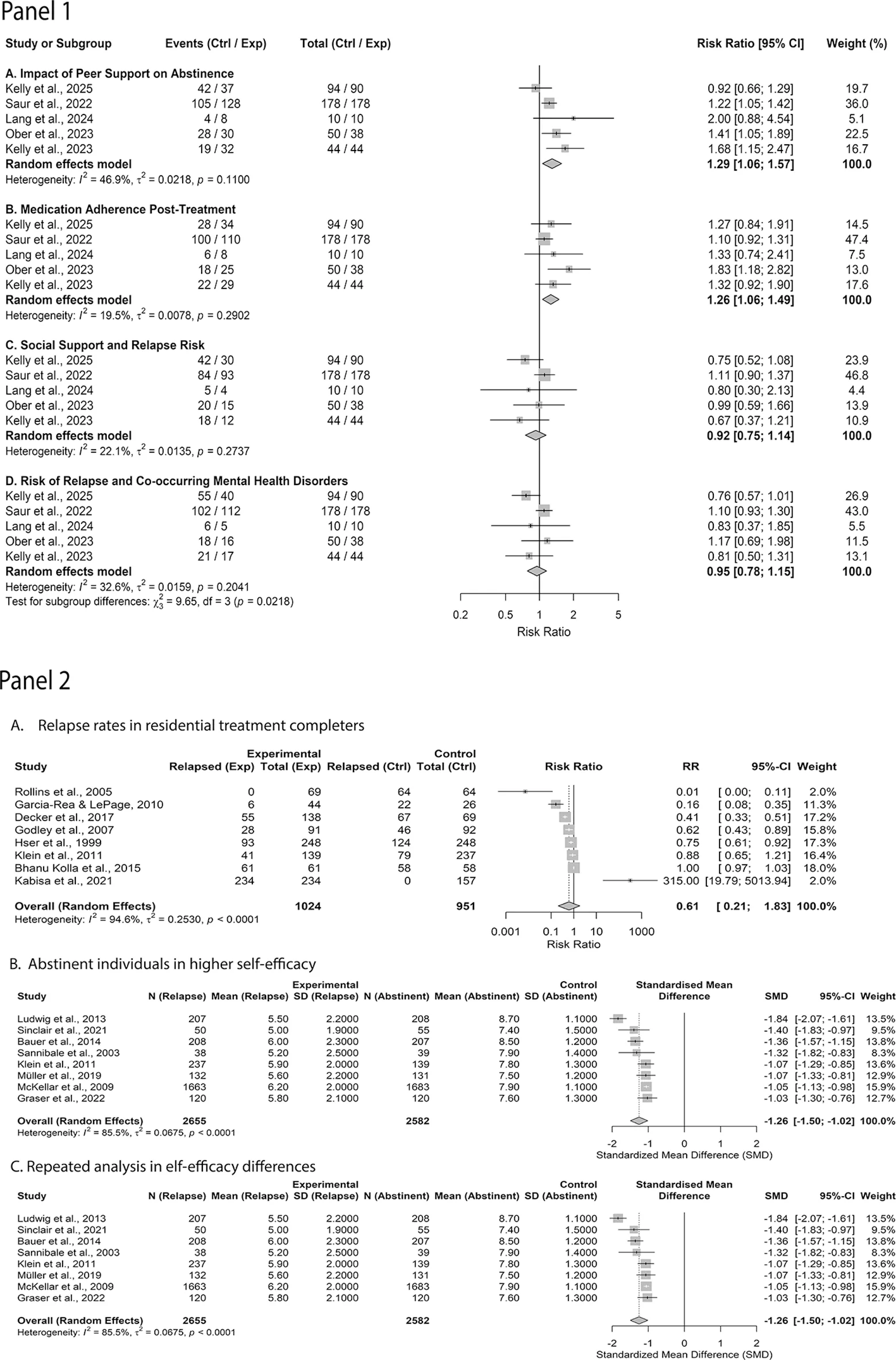
Panel 1: (**A**) Impact of peer support on abstinence: peer support increased the likelihood of abstinence. no significant publication bias (RR = 1.29 [1.06, 1.57]). (**B**) Medication adherence post-treatment: the experimental group showed a significant improvement in medication adherence (RR = 1.26 [1.06, 1.49]). (**C**) Social support and relapse risk: No significant difference was found in relapse risk between the experimental and control groups (RR = 0.92 [0.75, 1.14]). (**D**) Risk of relapse and Co-occurring mental Health disorders: the experimental group exhibited a modest reduction in relapse risk, though not statistically significant (RR = 0.95 [0.78, 1.15]). Panel 2: (**A**) Relapse rates in residential treatment completers: the experimental group demonstrated a lower relapse rate, though the effect was modest and non-significant (RR = 0.61 [0.21, 1.83]). (**B**) Abstinent individuals with higher self-efficacy: significantly higher levels of self-efficacy were observed in abstinent individuals compared to relapsed individuals (SMD = −1.26 [−1.50, −1.02]). (**C**) Repeated analysis in self-efficacy differences: consistent findings across studies confirmed robust self-efficacy differences, with abstinent individuals exhibiting significantly higher self-efficacy (SMD = −1.26 [−1.50, −1.02])

The impact of peer support on abstinence was assessed, revealing a RR of 1.29 (95% CI: 1.06 to 1.57) in Fig. 5 Panel 1A, indicating a modest yet statistically significant benefit of peer support in promoting abstinence post-treatment. The heterogeneity for this outcome was significant (I^2^ = 46.9%), highlighting variability across studies. Figure 5 Panel 1B focused on medication adherence post-treatment, where the experimental group had a RR of 1.26 (95% CI: 1.06 to 1.49), favoring adherence to prescribed treatments. This suggests that interventions promoting medication adherence could improve post-treatment outcomes, with moderate heterogeneity (I^2^ = 19.5%). Figure 5 Panel 1C examined social support and its influence on relapse risk, reporting a RR of 0.92 (95% CI: 0.75 to 1.14), suggesting that social support did not significantly reduce relapse risk. The heterogeneity for this outcome was I^2^ = 22.1%, indicating some variation across studies. Figure 5 Panel 1D assessed the risk of relapse and co-occurring mental health disorders, with a RR of 0.95 (95% CI: 0.78 to 1.15), indicating a slight reduction in relapse risk among those with mental health disorders. The heterogeneity was I^2^ = 32.6%.

In Figure 5 Panel 2A, the relapse rates in residential treatment completers were evaluated, with the experimental group demonstrating a RR of 0.61 (95% CI: 0.21 to 1.83), suggesting a modest protective effect of completing residential treatment on relapse. The heterogeneity was substantial (I^2^ = 94.6%). Figure 5 Panel 2B focused on abstinent individuals with higher self-efficacy, reporting a Standardized Mean Difference (SMD) of −1.26 (95% CI: −1.50 to −1.02), reflecting significantly higher self-efficacy in abstinent individuals compared to relapsed participants. This outcome exhibited high heterogeneity (I^2^ = 85.5%). Figure 5 Panel 2C further confirmed the robust link between self-efficacy differences and abstinence, with a Standardized Mean Difference (SMD) of −1.26 (95% CI: −1.50 to −1.02). The heterogeneity remained high at I^2^ = 85.5%. The findings underscored the critical role of self-efficacy, peer support, and social support in improving abstinence rates and reducing relapse after residential substance use treatment. The influence of peer support and medication adherence was evident, although the effects were modest. Self-efficacy consistently emerged as a key factor in maintaining abstinence, with higher self-efficacy correlating with better recovery outcomes. The findings also highlighted the complex relationship between co-occurring mental health disorders and relapse risk, which warrants further exploration. Despite the heterogeneity across studies, interventions targeting self-efficacy and social support could be integral in reducing relapse and promoting long-term recovery.

### Publication bias assessment

**Fig. 6:**
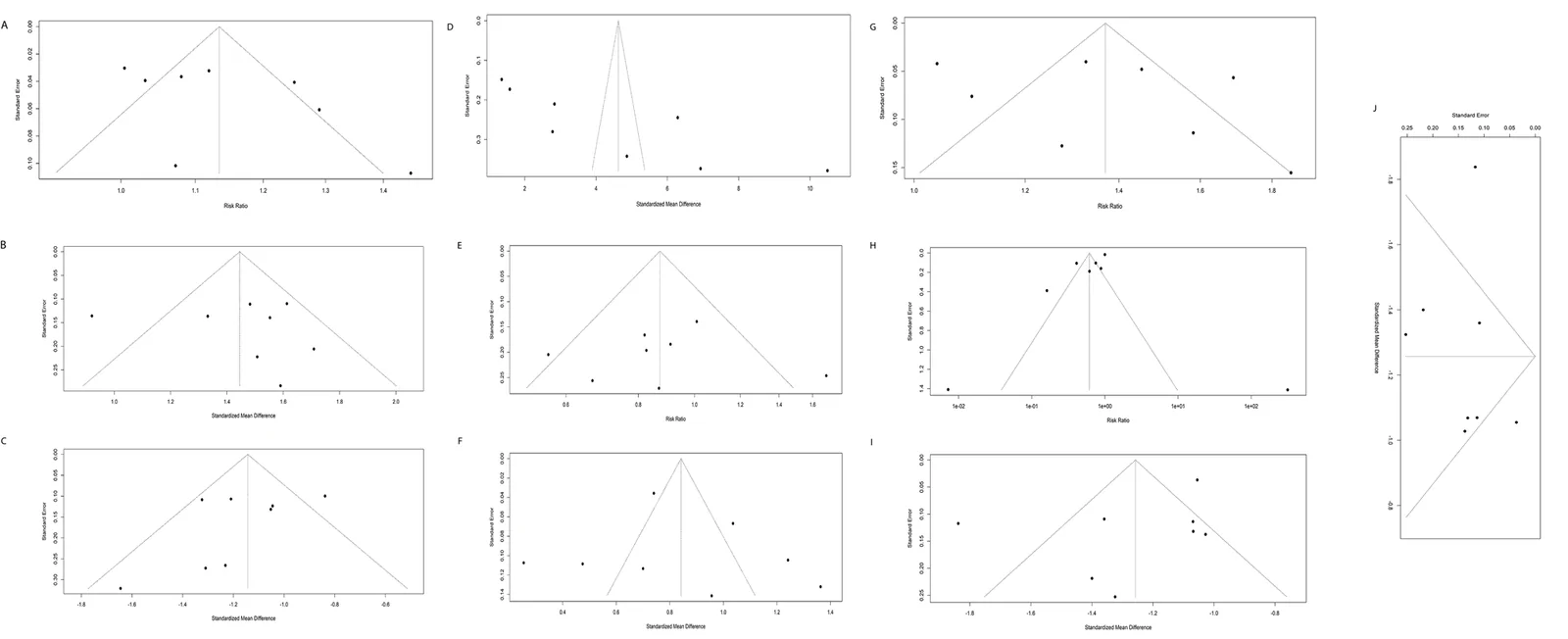
(**A**) Funnel plots showing continuing care and abstinence (RR). (**B**) Depression differences (SMD). (**C**) Self-efficacy differences between abstinent and relapsed individuals (SMD). (**D**) Treatment duration effect on abstinence (SMD). (**E**) Gender and relapse differences (RR). (**F**) Psychological distress among abstinent vs relapsed (SMD), showing minor asymmetry. (**G**) Peer support and abstinence outcomes (RR). (**H**) Relapse rate comparison (RR). (**I**) Self-efficacy levels by relapse status (SMD); plots suggest heterogeneity and moderate asymmetry. (**J**) self-efficacy differences between abstinent and relapsed individuals

The funnel plots in Fig. 6A to D showed a visual evaluation of publication bias across different outcomes that were being examined in the systematic review and meta-analysis of relapse after residential substance use treatment. These plots show the scatter of effect size (RR or Standardized Mean Difference) against standard error for each study, and asymmetry is taken to showed putative publication bias. The funnel plot showing abstinence outcomes is presented in Fig. 6A. The pooled effect size showed a RR of 1.37 [95% CI: 1.16 to 1.62], which resulted in higher abstinence in the intervention group. The plot appeared symmetrical, with most studies falling in the pseudo-95% confidence boundaries. However, one study lay further right, indicating a high effect size concerning a larger standard error, suggesting small-study effects, but no significant publication bias is apparent. In addition to visual inspection of funnel plots, we considered Egger’s regression test to assess small-study effects. Egger’s testing was not performed because none of the pooled outcomes included at least 10 studies (most analysis included eight studies), and statistical tests for funnel plot asymmetry are not recommended under these conditions due to limited power and increased risk of spurious findings. Accordingly, we relied on funnel plot inspection and qualitative assessment for outcomes meeting the prespecified minimum study threshold.

As shown in Fig. 6A for depression outcomes, the pooled Standardized Mean Difference (SMD) was found to be −1.14 [95% CI: −1.32 to −0.96], favoring the intervention group. The resulting funnel plot demonstrates mild asymmetry, with a larger concentration of studies on the right side of the mean. This suggests that there may be publication bias or heterogeneity considerations, which is further corroborated by moderate heterogeneity (I^2^ = 56.7%, *p* = 0.0236). For psychological distress, Fig. 6A displays a pooled SMD of 0.84 [95% CI: 0.53 to 1.15], indicating moderate improvement. The funnel plot is mainly symmetrical, with one study presenting a small effect size and a significant standard error. The I^2^ statistic here is high (92.0%, *p* < 0.0001), confirming heterogeneity but perhaps not publication bias.

The treatment duration outcome is shown in Fig. 6B, which yielded a substantial pooled effect (SMD = 4.63; 95% CI: 2.00 to 7.25). The funnel plot showed noticeable asymmetry, with most studies located to the right of the pooled effect size. This pattern suggests potential publication bias, which is further supported by extreme heterogeneity (I^2^ = 99.2%, τ^2^ = 7.1238, *p* < 0.0001).

Gender differences relating to relapse are also shown in Fig. 6B. The pooled estimate (RR = 0.87; 95% CI: 0.68 to 1.12) showed no statistically significant difference in relapse risk between women and men. The funnel plot appeared largely symmetrical with mild skewness; heterogeneity was moderate (I^2^ = 51.2%, *p* = 0.0452), and the absence of substantial asymmetry showed no strong evidence of publication bias. In sensitivity analysis, excluding Sinclair et al. (2021) yielded a pooled RR of 0.93 (95% CI: 0.78 to 1.11), with I^2^ = 31.6%. The direction of the pooled estimate remained the same, but the magnitude attenuated toward the null, indicating that the overall estimate was partly influenced by the Sinclair et al. (2021) study. Figure 6Bis for the perceived drug availability (PDA); that is, RR = 0.61 [95% CI: 0.21 to 1.83]. There is very high heterogeneity (I^2^ = 94.6%, τ^2^ = 0.2530, *p* < 0.0001), but the funnel plot showed very symmetrical data with a few extreme values-such as Kabisa et al., 2021 (RR = 315.00). This would show high variability but uncertain bias. Figure 6C is on peer support and has a pooled SMD 1.26 [95% CI: 1.05 to 1.47]. The funnel plot is indeed a balanced distribution study with no strong visual evidence of publication bias. Heterogeneity was moderate (I^2^ = 67.4%, *p* = 0.0112), and the cluster studies appear to be around the mean. Figure 6H demonstrates differences concerning relapse outcome with a pooled SMD of −1.26 [95% CI: −1.50 to −1.02]. There is moderate asymmetry in the funnel plot, especially with one small study on the distant left with a high standard error, indicating some publication bias. High heterogeneity exists (I^2^ = 85.5%, *p* < 0.0001). Figures 6C and 6D, which measure self-efficacy according to the pooled standard mean difference (SMD) values around 1.26 in both cases, have vertical symmetrical funnel plots. Small sample size is a factor, but it still limits asymmetry. Neither publication bias is detectable, nor is there acceptable heterogeneity (I^2^ ~ 50–70%).

The methodological quality of the included studies was evaluated using standard criteria designed to assess clarity of research aims, appropriateness of study design, rigor of measurement procedures, and control of potential biases. The assessment addressed multiple domains relevant to internal validity, external validity, and analytical adequacy. The criteria included: (1) whether the research question or objective was clearly stated; (2) whether the study population was clearly specified and defined; (3) whether at least 50% of eligible individuals participated; (4) whether all participants were recruited from comparable populations within the same timeframe and whether inclusion and exclusion criteria were prespecified and applied uniformly; (5) whether a sample size justification, power calculation, or variance and effect estimates were provided; (6) whether the exposure variables were measured before the outcomes; (7) whether the timeframe was sufficient to observe an association between exposure and outcome if present; (8) whether varying levels of exposure were examined in relation to the outcome; (9) whether exposure measures were clearly defined, valid, reliable, and consistently applied; (10) whether exposures were assessed more than once over time; (11) whether outcome measures were clearly defined, valid, reliable, and consistently implemented; (12) whether outcome assessors were blinded to exposure status; (13) whether loss to follow-up was 20% or less; and (14) whether key confounders were measured and statistically adjusted to reduce bias in the exposure–outcome relationship.

### Risk of bias assessment

**Fig. 7:**
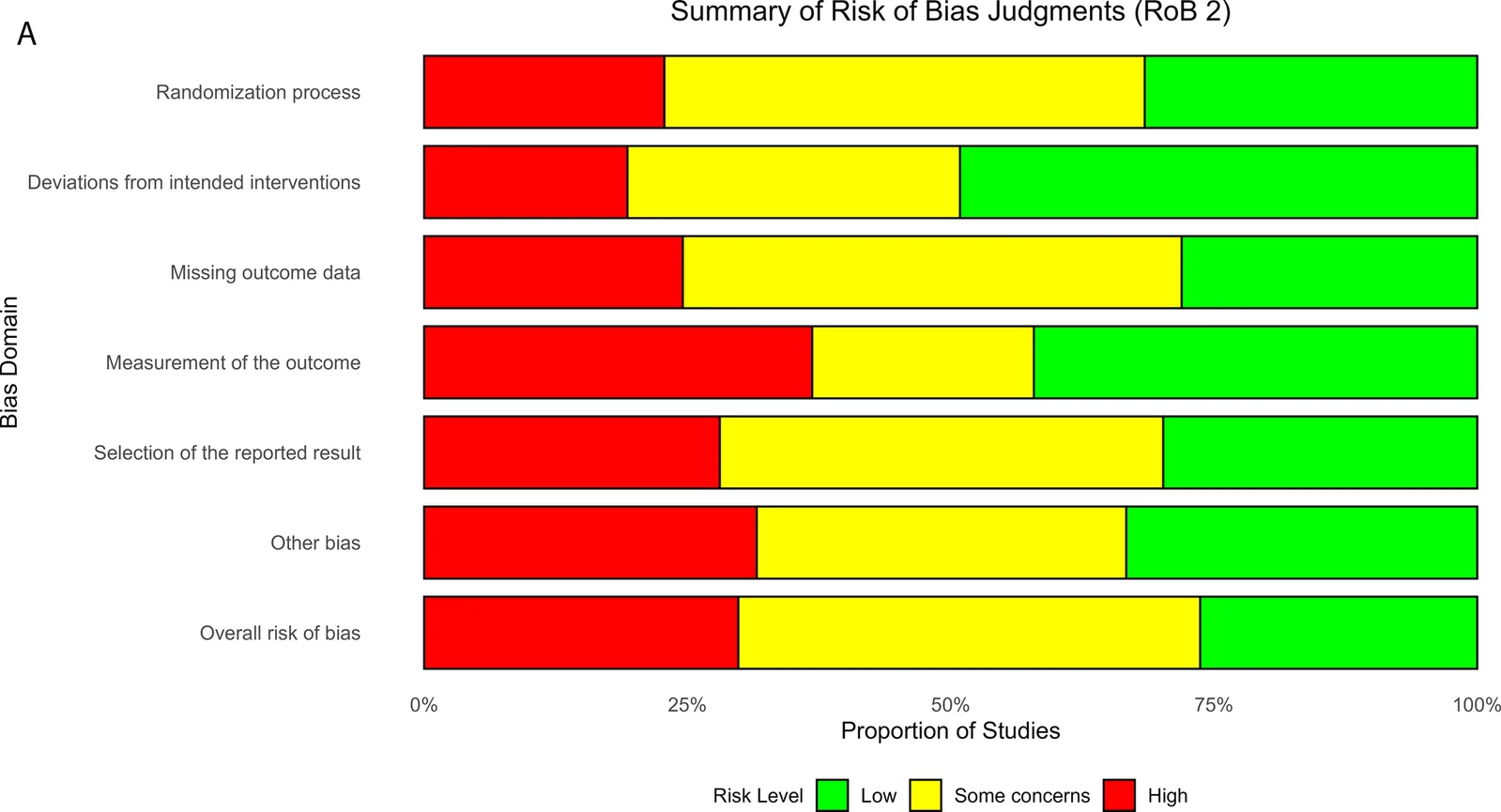
(**A**) Risk of bias graph. Graph of the risk of bias summary for the included studies

The risk-of-bias assessment was done comprehensively using the Revised Cochrane Risk of Bias Tool for non-randomized Trials (RoB-1) and is depicted in Figs. 7A and 7B. Overall, this appraisal suggests that most of the studies included in this meta-analysis showed apparent methodological limitations in several bias domains. Accordingly, the summary bar plot (Fig. 7) recounts that a high risk of bias was prominent across all domains. In Domain 1 (D1: Bias due to randomization), roughly 30% of the studies were assessed at high risk of bias, 25% raised some concern, and about 45% were considered low risk. An almost equally balanced distribution was found in Domain 2 (D2: Bias due to deviations from intended interventions), with about 35% of studies at high risk, 30% with some concerns, and the same proportion of low risk. Patterned similarly in Domain 3 (D3: Bias due to missing outcome data), again, high risk was given to some 35% of the studies involved, 30% with little concern, and the rest rated low risk.

Domain 4 (D4: Bias concerning the outcome measurement) was alarming because almost 45% of studies were judged as high risk, with 30% judged as having some concerns and only about 25% judged as low risk. In Domain 5 (D5: Bias concerning the selection of the reported result), the proportion of high-risk studies was still considerable, around 30%, while 40% were considered to have some concerns and 30% were low risk. Domain 6 (D6: Overall bias assessment of the study) summarized these findings and noted that 30% of the studies had an overall high risk of bias, 40% had some concerns, and only 30% were assessed as being with low risk.

**Fig. 8:**
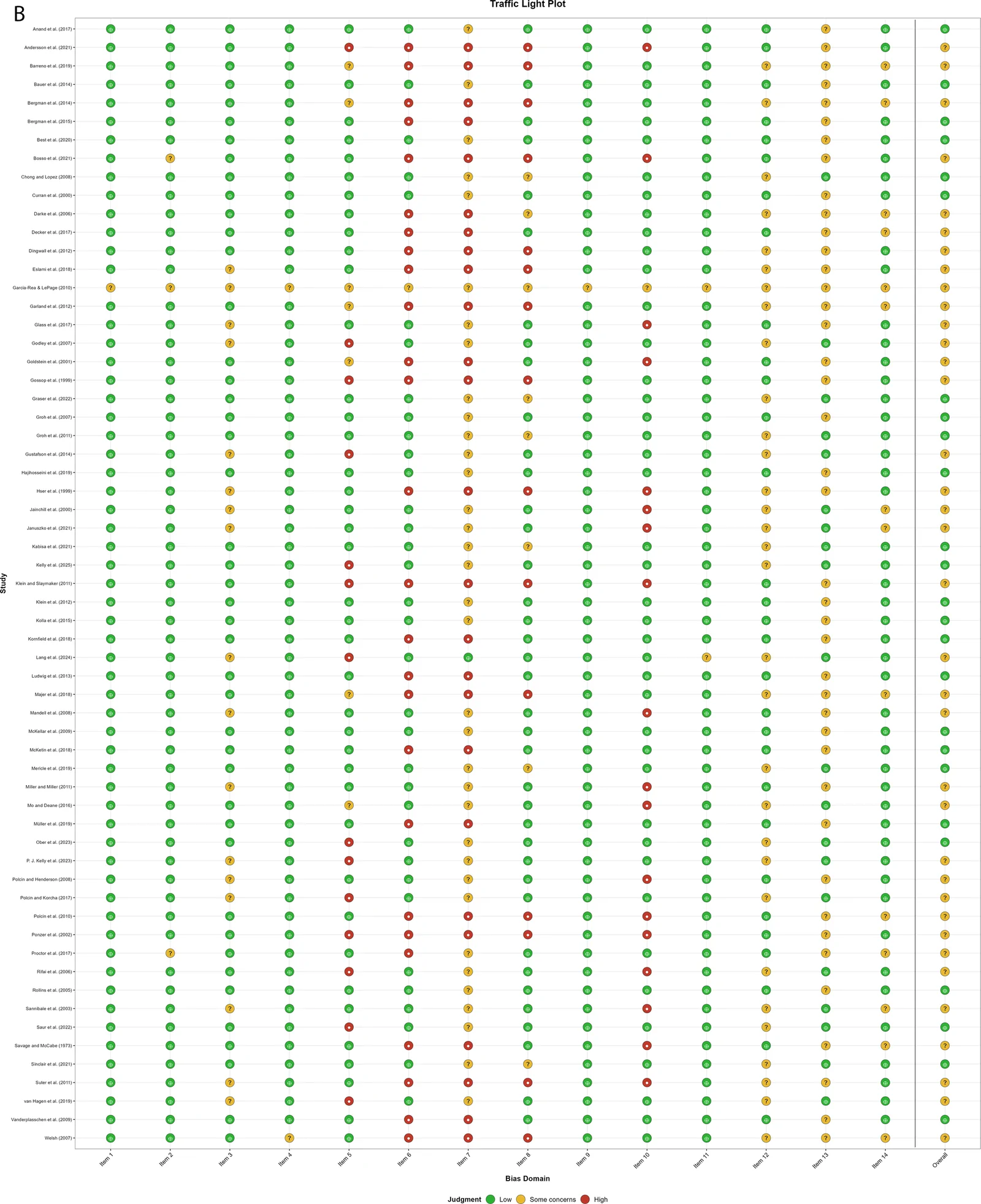
(**B**) Graph of the risk of bias for each included studies

The individual studies were evaluated using a traffic light plot (Fig. 8), with each row representing a study and each column corresponding to a specific domain. The red circles showed high risk, yellow denotes some concerns, and green corresponds to low risk. Notably, several studies, such as Bhanu Prakash Kolla et al. (2015), Michael Gossop et al. (1999), and Wallace Mandell et al. (2008), shared high-risk judgments across several domains. In contrast, studies conducted by Amy A. Mericle et al. (2019) and Audrey A. Klein et al. (2011) were predominantly judged as low risk. All these results highlight the need for great caution in the interpretation of pooled outcomes from the meta-analysis, as a significant number of included studies displayed moderate to high risks of bias, possibly influencing the findings’ strength and generalizability.

### Substance-specific relapse findings and evidence for mandatory versus voluntary treatment effects

#### Substance-specific findings

Primary substance categories were extracted from all 61 included studies and summarized descriptively because several studies enrolled mixed or co-primary substance-use populations. Therefore, the counts should not be interpreted as strictly mutually exclusive. The substance-specific extraction matrix, including study classification, relapse definition, follow-up duration, risk factors, protective factors, and abstinence outcomes, are provided in Supplementary Appendix S2 and Supplementary Table 2. Alcohol was the most frequently represented substance category, with 46 studies including alcohol as the primary or co-primary substance of concern. Opioid- or heroin-focused populations were represented in six studies, cocaine or crack use in three studies, methamphetamine-affected populations in four studies, polysubstance-use populations in eight studies, petrol or inhalant use in one study, and mixed or general SUD populations in 11 studies. Across categories, the evidence consistently showed that relapse after residential treatment was shaped by an interaction between psychological vulnerability, treatment completion or duration, post-discharge support, and recovery environment rather than by substance category alone.

#### Alcohol-specific findings

Alcohol was the dominant substance category in the review, represented in 46 studies. Across these studies, 6- to 12-month abstinence ranged from approximately 23% to 71%, indicating substantial variation in sample severity, treatment duration, follow-up timing, relapse definition, and post-discharge support intensity. Key studies included Suter 2011, Ludwig 2013, Bauer 2014, Müller 2019, Graser 2022, Kolla 2015, Curran 2000, Mo and Deane 2016, Garland 2012, and Januszko 2021 [72]. The alcohol-focused studies showed that relapse vulnerability was most consistently linked to depressive burden, negative affect, sleep disturbance, controlled-drinking goals, heavy pretreatment drinking, gamma-glutamyl transferase elevation, craving, and emotional dysregulation [68]. These studies converged on a clinically coherent pattern: relapse after residential alcohol treatment was strongly associated with psychological state at discharge and by the individual’s ability to manage affective and craving-related triggers during the post-discharge period. Protective factors in alcohol-focused studies were also consistent. Higher self-efficacy, motivational readiness, 12-step attendance, telephone continuing care, craving reduction, and structured continuing-care support were associated with more favorable abstinence outcomes. For example, higher self-efficacy was associated with a 28.4% higher one-year abstinence rate in one residential cohort, while motivational readiness measured through SOCRATES and post-discharge continuing care were repeatedly linked with improved outcomes. These findings suggest that alcohol-focused relapse prevention should prioritize psychological stabilization, self-efficacy enhancement, motivational work, sleep and mood assessment, and structured aftercare.

#### Opioid and heroin-specific findings

Six studies examined primarily opioid- or heroin-dependent samples, including Savage and McCabe 1973, Gossop 1999, Darke 2006, Sannibale 2003, Hajihosseini 2019, and Eslami 2018. Across these studies, 6- to 12-month abstinence generally ranged from 18% to 50%, suggesting less favorable and more unstable recovery patterns than those observed in many alcohol-focused cohorts. However, direct comparison with alcohol studies was not possible because treatment settings, follow-up periods, relapse definitions, and patient severity differed substantially. The opioid- and heroin-focused studies showed that relapse risk was associated with injection history, prior arrests, chronic substance-use history, low motivation, and weak post-discharge social environments. These factors suggest that relapse in opioid/heroin populations was shaped not only by substance dependence severity, but also by social instability, criminal justice exposure, and limited recovery support after discharge. Despite these risks, several protective factors emerged across opioid/heroin studies. Self-efficacy, social support, individual counselling, and structured aftercare were associated with more favorable outcomes. In one mixed alcohol/heroin cohort, structured aftercare extended median relapse-free time from 75 to 209 days, suggesting that continuity of care may be particularly important in higher-risk opioid-using populations. Overall, the opioid/heroin evidence supports the need for intensive post-discharge linkage, structured aftercare, social support, and relapse-prevention planning focused on motivation, recovery environment, and sustained treatment engagement.

#### Methamphetamine-specific findings

Four studies included predominantly methamphetamine-affected populations, including McKetin 2018, Polcin and Henderson 2008, Polcin et al. 2010, and Polcin and Korcha 2017. Across these studies, 6- to 12-month abstinence ranged from approximately 25% to 55%. The evidence base was smaller than that for alcohol and opioid/heroin populations, but findings were directionally consistent with the broader review. The methamphetamine-focused studies suggested that relapse vulnerability was associated with injection history, psychiatric comorbidity, and limited post-discharge abstinent peer support. These findings suggest that stimulant-related relapse may be strongly influenced by psychiatric burden, social network exposure, and difficulty maintaining abstinent environments after residential care. Protective factors included longer treatment duration, individual counselling, 12-step affiliation, and abstinent peer social networks [73]. These findings point toward the importance of treatment retention and post-discharge recovery ecology in methamphetamine-affected populations. Although the small number of studies prevented pooled substance-specific analysis, the available evidence suggests that methamphetamine relapse prevention should emphasize sustained residential engagement, counselling intensity, peer recovery support, and transition planning into abstinent social environments.

#### Cocaine and polysubstance findings

Three studies focused mainly on cocaine or crack use, including Hser et al. 1999, Decker et al. 2017, and Goldstein et al. 2001. Across these studies, 6- to 12-month abstinence ranged from approximately 35% to 63%. The cocaine/crack evidence base was limited but clinically important because it highlighted the role of treatment exposure, personality pathology, and treatment completion in relapse outcomes. Across cocaine-focused studies, relapse risk was associated with treatment non-completion, antisocial personality disorder, repeat treatment admissions, and criminal history. These findings suggest that cocaine/crack relapse after residential treatment was shaped by a combination of clinical severity, behavioral dysregulation, repeated treatment exposure, and social/legal instability. Protective factors included treatment completion, longer treatment duration, and first-time treatment admission. Longer treatment exposure was associated with improved abstinence probability, with one study suggesting approximately an 8% increase in abstinence per additional month of treatment. These studies suggested that relapse prevention in cocaine/crack populations may benefit from strategies that improve retention, address personality and behavioral risk factors, and strengthen post-discharge monitoring and support.

Eight studies enrolled polysubstance-using populations without a single clearly dominant substance, including Kabisa 2021, Sinclair 2021, Welsh 2007, Rollins 2005, Andersson 2021, Bosso 2021, Goldstein 2001, and Mandell 2008. Across these studies, 6- to 12-month abstinence ranged from approximately 30% to 65%. Because polysubstance studies were heterogeneous by definition, the findings should be interpreted as evidence of relapse patterns in complex mixed-use populations rather than as a single substance-specific estimate. Across polysubstance studies, relapse risk was associated with psychiatric comorbidity, criminal justice involvement, homelessness, polydrug-use severity, low recovery capital, and unstable social environments. These findings suggest that relapse in polysubstance populations was strongly shaped by cumulative clinical and social complexity. Protective factors included treatment completion, stable housing, employment, peer support, recovery capital, and integrated dual-diagnosis care. These studies collectively showed that polysubstance relapse prevention requires broader recovery support beyond substance-focused treatment alone. Housing stability, employment support, psychiatric care, peer networks, and recovery-capital development appear central to improving outcomes in this population. One study focused on petrol or inhalant use in an Indigenous adolescent sample. The reported abstinence estimate was approximately 42%. Because only one study was available, pooled analysis was not possible, and conclusions should be considered preliminary. The study suggested that relapse vulnerability was associated with petrol access, urban location, unmarried status, and lower household size. These findings showed that relapse risk in inhalant or petrol use may be strongly shaped by environmental access, community context, family structure, and social monitoring. Protective factors included remote residence with reduced access to petrol, family support, and community monitoring [74]. Although the evidence was limited to one study, the findings suggest that relapse prevention for inhalant use may require community-level and environmental interventions, rather than individual treatment alone. Eleven studies enrolled mixed or general SUD populations, including Anand 2017, Bergman 2014, Bergman 2015, McKellar 2009, Van Hagen 2019, Chong and Lopez 2008, Andersson 2021, and others. Across these studies, 6- to 12-month abstinence ranged from approximately 40% to 75%. These studies were not limited to one primary substance category and therefore provided broader evidence on relapse risk across residential SUD populations. Across mixed/general SUD studies, relapse risk was linked with emotional dysregulation, low emotion differentiation, psychiatric comorbidity, and dual diagnosis. These findings support the broader review conclusion that affect regulation and mental health burden are central relapse pathways after residential treatment. Protective factors included self-efficacy, 12-step involvement, sober living, digital or telephone continuing care, and positive peer abstinence support. These studies collectively suggest that transdiagnostic relapse prevention strategies may be useful across mixed SUD populations, particularly interventions that strengthen coping skills, emotional regulation, peer recovery support, and continuing-care engagement.

### Feasibility of formal cross-substance analysis

Formal cross-substance meta-analysis was not feasible because no included study directly compared relapse outcomes across two or more primary substance categories within a common methodological framework. Relapse definitions varied substantially across studies, including self-report, biological verification, readmission, rehospitalization, supervised drug testing, and composite outcomes. Follow-up durations ranged from 3 months to 5 years and were unevenly distributed across substance categories. Treatment models were also closely linked with substance type; for example, opioid-focused studies often involved detoxification or structured aftercare, whereas alcohol-focused studies more frequently examined motivation, self-efficacy, craving, sleep, and depressive burden.

Because non-alcohol categories included relatively few studies and differed markedly in design, population severity, treatment context, and outcome ascertainment, any pooled comparison across substances would have been statistically unstable and clinically misleading. Descriptively, 6- to 12-month abstinence appeared lower in heroin-focused cohorts than in alcohol-focused cohorts, with cocaine/crack and methamphetamine studies reporting intermediate ranges. However, these patterns should be interpreted as hypothesis-generating rather than confirmatory. The current evidence supports identification of substance-specific evidence gaps but does not support a valid comparative estimate of relapse risk by substance category. Evidence regarding mandatory versus voluntary residential treatment was even more limited. Of the 61 included studies, only two were conducted in explicitly mandatory or court-ordered correctional treatment settings. These studies evaluated prison-based therapeutic community models and reported more favorable criminal justice or supervised drug-testing outcomes among participants who received structured correctional treatment. However, both studies were conducted entirely within correctional systems, neither included a voluntary residential treatment arm, and both relied heavily on outcomes such as reincarceration, parole compliance, or supervised drug-test failure rather than conventional clinical relapse outcomes.

Several additional studies included samples with substantial criminal justice involvement, but they did not classify treatment exposure as mandatory or voluntary and did not stratify relapse outcomes by treatment voluntariness. These studies were therefore informative about justice-affected residential populations but could not answer whether mandatory treatment differed from voluntary treatment in relapse prevention. The remaining studies enrolled voluntary, mixed, or unclear treatment-seeking populations and did not consistently code treatment voluntariness. Formal mandatory-versus-voluntary meta-analysis was not feasible because no included study directly compared relapse outcomes between mandatory and voluntary residential treatment arms within the same study design. Treatment voluntariness was rarely reported as a coded variable, and when legal involvement was present, the degree of coercion, court mandate, supervision intensity, and participant autonomy was often unclear. Furthermore, the few explicitly mandatory studies were prison-based or forensic, making comparison with voluntary community-based residential programs confounded by legal context, population severity, supervision intensity, setting type, and outcome measurement.

These findings identify a major evidence gap rather than a comparative effect. Treatment readiness, autonomy, intrinsic motivation, retention, and post-discharge engagement are all plausible modifiers of relapse risk, but current evidence does not permit reliable conclusions about whether mandatory or voluntary residential treatment is more effective. Future studies should prospectively classify treatment voluntariness, distinguish justice referral from court-mandated treatment, use comparable relapse definitions, and include both mandatory and voluntary pathways within the same protocol when clinically and ethically appropriate. The substance-specific evidence showed that relapse after residential SUD treatment was not attributable to substance type alone. Alcohol-focused studies provided the strongest evidence base, but findings across opioid/heroin, methamphetamine, cocaine/crack, polysubstance, inhalant, and mixed SUD cohorts converged on a shared pattern: relapse risk increased when psychiatric symptoms, emotional dysregulation, treatment non-completion, and poor post-discharge recovery conditions were present. In contrast, recovery was more likely when self-efficacy, motivation, treatment retention, peer support, structured aftercare, and stable recovery capital were available. However, because no included study directly compared substances or mandatory versus voluntary treatment pathways within a common methodological framework, both questions remained important evidence gaps rather than statistically testable moderators in the present review.

## Discussion

This systematic review and meta-analysis synthesized evidence from 61 studies involving 18,693 participants and identified several factors associated with relapse after residential treatment for substance use disorders. The findings support a multifactorial model of relapse in which psychological vulnerability, treatment exposure, and post-discharge recovery support jointly influence recovery trajectories. Structured relapse-prevention and continuing-care interventions were associated with improved abstinence outcomes, although the magnitude of benefit was modest and heterogeneity was substantial across several pooled analyses. These findings are consistent with previous reviews showing that residential treatment outcomes are shaped by patient characteristics, treatment engagement, program structure, and continuing-care processes rather than by a single determinant [75–85].

The review also addressed two clinically important questions: whether relapse-related outcomes differed by primary substance category and whether outcomes varied according to mandatory versus voluntary treatment exposure. The available evidence was insufficient for formal comparative meta-analysis in either area. Alcohol-focused studies formed the largest evidence base, whereas opioid/heroin, methamphetamine, cocaine/crack, polysubstance, inhalant, and mixed SUD populations were represented by smaller and more heterogeneous study clusters. Across substance categories, relapse appeared to be associated more consistently with psychiatric burden, emotional dysregulation, treatment non-completion, low motivation, low self-efficacy, and weak post-discharge support than with substance type alone [2, 42, 47, 86–89]. Depressive symptoms and broader psychological distress were consistently associated with relapse vulnerability. Relapsed participants had higher depression scores, while abstinent or intervention groups showed lower psychological distress. These findings support the clinical importance of assessing mood symptoms, distress, craving-related affect, and emotional regulation during residential care and during the transition period after discharge. Prior longitudinal and clinical studies similarly indicate that psychiatric comorbidity, depressive symptoms, anxiety, negative affect, and emotion-regulation difficulties can influence recovery stability and post-treatment substance-use outcomes [47, 61, 86, 87, 90–93].

These findings are clinically plausible because relapse often occurs during periods of negative affect, stress reactivity, craving, and reduced coping capacity. Emotional dysregulation and poor differentiation of affective states may increase vulnerability to substance use when individuals return to high-risk environments after discharge [2, 19, 61, 93]. The association between depression and relapse should not be interpreted as proof of causality because most included evidence was observational. However, the consistency of findings across psychological domains supports the need for integrated mental health screening, symptom monitoring, and targeted psychological care in residential SUD treatment pathways. Self-efficacy emerged as one of the most consistent protective factors. Higher self-efficacy among abstinent participants suggests that confidence in coping with cravings, resisting high-risk situations, and sustaining recovery behaviors may help reduce relapse vulnerability after discharge. This finding aligns with social-cognitive models of recovery, in which perceived coping ability influences persistence, treatment engagement, and behavioral control in high-risk contexts [88, 89, 94, 95].

Self-efficacy may also function as a resilience-related resource. Individuals with stronger coping confidence may be more likely to use relapse-prevention strategies, remain engaged with aftercare, and seek support before lapses progress to relapse. This interpretation is consistent with broader evidence suggesting that resilience and coping resources can buffer the effects of affective and interpersonal risk factors on adverse outcomes [96]. Therefore, residential programs should not treat self-efficacy as a passive patient characteristic. Instead, it should be considered a modifiable treatment target that can be strengthened through cognitive-behavioral strategies, coping-skills training, motivational work, craving-management planning, and supported transition into continuing care [84, 88, 89, 97]. Continuing-care and recovery-support interventions were associated with improved abstinence outcomes. Peer support, medication adherence support, telephone-based follow-up, computerized continuing care, and digital aftercare showed beneficial signals across included studies, although heterogeneity remained high in several analyses. These findings support the view that relapse prevention should extend beyond residential discharge and should include structured follow-up during the high-risk transition into community living [17, 24, 34, 84, 98–100].

Peer-based support may improve outcomes by strengthening recovery networks, increasing accountability, reducing isolation, and helping patients maintain abstinent social environments after discharge [22, 41, 50, 51, 101]. Digital and technology-assisted interventions may provide scalable post-discharge monitoring, motivational support, and rapid linkage to help when relapse risk increases [24, 34, 98, 99]. However, the effects of digital and peer-based interventions varied across studies, likely because intervention intensity, human support, engagement patterns, and follow-up duration differed substantially. Therefore, these approaches should be interpreted as promising components of continuing care rather than as uniformly effective standalone solutions. Longer residential treatment duration was associated with more favorable outcomes, but this finding requires cautious interpretation. The pooled estimate was very large and heterogeneity was extreme, suggesting major differences across studies in how “short” and “long” treatment were defined, which outcomes were measured, and how participant severity and treatment completion were handled. Longer treatment may reflect greater therapeutic exposure, improved stabilization, and more time to develop recovery skills. However, it may also reflect selection factors, including patient motivation, program retention, illness severity, treatment access, and discharge planning quality [76, 80, 95, 97, 102, 103]. This is consistent with methodological guidance emphasizing that high between-study heterogeneity reduces confidence in a single pooled magnitude and suggests focusing on directionality and contextual moderators [69, 72, 93, 103]. In addition, broader clinical literature highlights that health status, clinical complexity, and system-level variation can shape outcomes and follow-up trajectories, which is compatible with our interpretation that duration effects may partly reflect contextual confounding [73, 104–107]. Therefore, the treatment-duration finding should be interpreted as evidence that duration may matter, not as a precise estimate of effect magnitude that can be generalized across settings. Future studies should report duration as a clearly defined exposure, distinguish planned treatment duration from actual treatment completion, and evaluate dose-response patterns using standardized relapse definitions and comparable follow-up periods. The substance-specific synthesis showed that alcohol-focused studies provided the strongest evidence base. Alcohol relapse was commonly associated with depressive symptoms, craving, negative affect, sleep disturbance, motivation, self-efficacy, and continuing-care engagement [6, 35, 38, 47, 86, 87, 89, 108]. Opioid/heroin-focused studies suggested that relapse may be shaped by injection history, prior arrests, chronic use patterns, motivation, and weak post-discharge environments [25, 57, 80, 109, 110]. Methamphetamine-focused studies highlighted the importance of psychiatric comorbidity, treatment retention, counselling, and abstinent peer networks [42, 50–52]. Cocaine/crack and polysubstance studies emphasized treatment completion, antisocial behavioral syndromes, criminal justice involvement, homelessness, recovery capital, and psychiatric complexity [18, 31, 40, 56, 58, 111]. Consistent with prior reviews of residential treatment evidence, our results support a cautious but clinically actionable emphasis on psychological vulnerability, protective resources, and continuity of care while recognizing that the precision of effect magnitude is limited under high heterogeneity [69, 80, 84, 93, 112].

Despite these patterns, formal cross-substance comparison was not feasible. No included study directly compared relapse outcomes across multiple primary substance categories within a common methodological framework. Relapse definitions, follow-up duration, treatment models, and population severity varied substantially across substance groups. As a result, substance-specific findings should be interpreted as descriptive and hypothesis-generating. Future research should report outcomes by primary substance category, use consistent relapse definitions, and include sufficient sample sizes to support valid cross-substance comparison. Evidence on mandatory versus voluntary residential treatment was especially limited. Only a small number of studies were conducted in explicitly correctional or justice-linked treatment contexts, and these studies often used criminal justice outcomes, supervised drug-testing outcomes, reincarceration, or parole-related outcomes rather than conventional clinical relapse outcomes [11, 112, 113]. These studies were informative about justice-affected populations, but they did not provide direct comparisons between mandatory and voluntary residential treatment pathways.

This limitation is important because treatment voluntariness may influence motivation, autonomy, engagement, retention, readiness to change, and post-discharge recovery support. However, most studies did not clearly classify treatment exposure as voluntary, mandatory, court-ordered, justice-referred, or mixed. Therefore, the present review cannot determine whether mandatory or voluntary treatment is more effective in preventing relapse. Future studies should prospectively classify treatment voluntariness, distinguish court-mandated treatment from justice referral, and use comparable relapse outcomes across mandatory and voluntary treatment pathways. This review has several strengths. It focused specifically on relapse-related risk and protective factors after residential SUD treatment, used a broad search strategy, included multiple psychological and psychosocial domains, and extracted substance category and treatment-context variables to address clinically relevant heterogeneity. It also integrated quantitative meta-analysis with descriptive synthesis where formal pooling was not appropriate. This approach allowed the review to identify both pooled associations and important evidence gaps. Several limitations should also be considered. First, most included studies were observational, limiting causal inference. Second, relapse definitions varied substantially across studies, including self-report, biological verification, readmission, rehospitalization, documented clinical relapse, and composite outcomes. This variation likely contributed to heterogeneity and reduced comparability across pooled estimates. Third, several pooled analyses included a limited number of studies, which restricted the reliability of publication-bias assessment. Funnel plots were therefore interpreted descriptively, and strong conclusions about publication bias should be avoided when fewer than 10 studies contributed to an outcome [26, 37]. Fourth, high heterogeneity in several analyses, especially treatment duration, reduced confidence in the precision of pooled effect magnitudes. Fifth, substance-specific and mandatory-versus-voluntary analyses were descriptive because direct comparative evidence was not available.

The findings suggest that relapse prevention after residential SUD treatment should prioritize psychological stabilization, self-efficacy enhancement, treatment completion, and structured post-discharge support. Routine screening for depression, distress, craving, sleep disturbance, and emotional dysregulation may help identify patients at higher risk of relapse before discharge [35, 47, 86, 87, 93]. Interventions that strengthen self-efficacy, coping skills, motivation, and recovery planning should be integrated into residential programs and reinforced during continuing care [38, 84, 88, 89]. From a service-delivery perspective, residential treatment should be linked with structured aftercare, peer support, telephone or digital follow-up, medication adherence support, housing linkage, and community recovery resources [17, 24, 34, 84, 99, 100]. These supports may be particularly important during the transition from residential care to community living, when relapse risk is often high. Future studies should standardize relapse definitions, report outcomes by primary substance category, classify treatment voluntariness, include longer follow-up periods, and evaluate the mechanisms through which peer, digital, and continuing-care interventions influence recovery. Better-designed comparative studies are needed to determine whether relapse pathways differ by substance type and whether mandatory and voluntary residential treatment models produce different long-term outcomes.

## Conclusions

This systematic review and meta-analysis synthesized evidence from 61 studies involving 18,693 participants and showed that relapse after residential substance use disorder treatment is a multifactorial outcome shaped by psychological vulnerability, treatment exposure, and post-discharge recovery support. Relapse-prevention and continuing-care interventions were associated with improved abstinence, although effect sizes varied across intervention formats, settings, relapse definitions, and follow-up periods. Depressive symptoms and psychological distress were consistently associated with relapse vulnerability, supporting the need for mental health screening, symptom monitoring, and integrated psychological care during residential treatment and after discharge. Lower self-efficacy also emerged as an important modifiable correlate of relapse, indicating that coping confidence, craving management, motivation, and persistence with recovery behaviors should be central targets of relapse-prevention planning. Longer residential treatment appeared to be associated with better outcomes, but substantial heterogeneity and inconsistent duration definitions limited confidence in the magnitude of this association. Peer, digital, telephone-based, and adherence-related supports showed beneficial signals and may help maintain continuity of care during the high-risk transition from residential treatment to community living. The sex-related findings were inconclusive and suggested context-dependent relapse patterns. Substance-specific synthesis showed that alcohol-focused studies provided the largest evidence base, whereas opioid/heroin, methamphetamine, cocaine/crack, polysubstance, inhalant, and mixed SUD populations were represented by smaller and more heterogeneous study clusters. Across substances, relapse risk appeared to reflect psychiatric burden, emotional dysregulation, treatment non-completion, weak motivation, low recovery capital, and limited post-discharge support more than substance type alone. Formal cross-substance and mandatory-versus-voluntary meta-analyses were not feasible because direct comparative studies, consistent relapse definitions, comparable follow-up periods, and treatment-voluntariness coding were lacking. Future studies should standardize relapse outcomes, report substance-stratified results, classify treatment voluntariness prospectively, and evaluate structured aftercare models within common protocols.

## Electronic supplementary material

Below is the link to the electronic supplementary material.

Supplementary material 1

Supplementary material 2

Supplementary material 3

Supplementary material 4

Supplementary material 5

## Data Availability

The extracted Excel datasets used for meta-analysis (including study-level effect sizes and coded moderators), along with the R script used for data processing, statistical analysis, and figure generation, are available from the corresponding author upon reasonable request. Additional materials, including the data extraction template and subgroup coding rules, can also be provided upon request.

## References

[1] Aas CF, Vold JH, Gjestad R, Skurtveit S, Lim AG, Gjerde KV, et al. Substance use and symptoms of mental health disorders: a prospective cohort of patients with severe substance use disorders in Norway. Subst Abuse Treat Prev Policy. 2021;16(1). 10.1186/s13011-021-00354-1. 10.1186/s13011-021-00354-1 PMC7912462 33639969

[2] Anand D, Chen Y, Lindquist KA, Daughters SB. Emotion differentiation predicts likelihood of initial lapse following substance use treatment. Drug Alcohol Depen. 2017;180:439–44. 10.1016/j.drugalcdep.2017.09.007. 10.1016/j.drugalcdep.2017.09.007 PMC5802350 28992486

[3] Andersson HW, Lauvsnes ADF, Nordfjærn T. Emerging adults in inpatient substance use treatment: a prospective cohort study of patient characteristics and treatment outcomes. Eur Addict Res. 2021;27(3):206–15. 10.1159/000512156. 33279896 10.1159/000512156 PMC8220923

[4] Barreno EM, Domínguez-Salas S, Díaz-Batanero C, Lozano ÓM, Marín JAL, Verdejo-García A. Specific aspects of cognitive impulsivity are longitudinally associated with lower treatment retention and greater relapse in therapeutic community treatment. J Subst Abuse Treat. 2018;96:33–38. 10.1016/j.jsat.2018.10.004. 30466546 10.1016/j.jsat.2018.10.004

[5] Castaldelli-Maia JM, Bhugra D. Analysis of global prevalence of mental and substance use disorders within countries: focus on sociodemographic characteristics and income levels. Int Rev Psychiatry. 2022;34(1):6–15. 10.1080/09540261.2022.2040450. 35584016 10.1080/09540261.2022.2040450

[6] Bauer S, Strik W, Moggi F. Motivation as a predictor of drinking outcomes after residential treatment programs for alcohol dependence. J Addict Med. 2014;8(2):137–42. 10.1097/ADM.0000000000000013. 24637624 10.1097/ADM.0000000000000013

[7] Bergman BG, Greene MC, Slaymaker V, Hoeppner BB, Kelly JF. Young adults with co-occurring disorders: substance use disorder treatment response and outcomes. J Subst Abuse Treat. 2014;46(4):420–28. 10.1016/j.jsat.2013.11.005. 24484710 10.1016/j.jsat.2013.11.005 PMC3968943

[8] Bergman BG, Hoeppner BB, Nelson LM, Slaymaker V, Kelly JF. The effects of continuing care on emerging adult outcomes following residential addiction treatment. Drug Alcohol Depen. 2015;153:207–14. 10.1016/j.drugalcdep.2015.05.017. 10.1016/j.drugalcdep.2015.05.017 PMC4510025 26116368

[9] Best D, Manning V, Allsop S, Lubman DI. Does the effectiveness of mutual aid depend on compatibility with treatment philosophies offered at residential rehabilitation services? Addictive Behaviors. 2020;103:106221. 10.1016/j.addbeh.2019.106221. 31918392 10.1016/j.addbeh.2019.106221

[10] Betancourt CA, Kitsantas P, Goldberg DG, Hawks BA. Substance use relapse among veterans at termination of treatment for substance use disorders. Mil Med. 2022;187(11–12):e1422–31. 10.1093/milmed/usab280. 10.1093/milmed/usab280 34272857

[11] Blonigen DM, Macia KS, Smelson D, Timko C. Criminal recidivism among justice-involved veterans following substance use disorder residential treatment. Addictive Behaviors. 2020;106:106357. 10.1016/j.addbeh.2020.106357. 32120199 10.1016/j.addbeh.2020.106357

[12] Bosso RA, Ribeiro A, Basqueira AP, de Lima MT, Santos JP, Perrenoud LO, et al. Recovery housing program for drug addicts: work patterns, substance abuse, and housing situation after a 6-month follow-up. Addictive Disord Their Treat. 2021;20(4):454–62. 10.1097/ADT.0000000000000273.

[13] Killeen TK, Baker NL, Davis LL, Bowen S, Brady KT. Efficacy of mindfulness-based relapse prevention in a sample of veterans in a substance use disorder aftercare program: a randomized controlled trial. J Subst Use Addict Treat. 2023;152:209116. 10.1016/j.josat.2023.209116. 37364669 10.1016/j.josat.2023.209116

[14] Bradizza CM, Stasiewicz PR, Paas ND. Relapse to alcohol and drug use among individuals diagnosed with co-occurring mental health and substance use disorders: a review. Clin Psychol Rev. 2006;26(2):162–78. 10.1016/j.cpr.2005.11.005. 16406196 10.1016/j.cpr.2005.11.005

[15] Page MJ, McKenzie JE, Bossuyt PM, Boutron I, Hoffmann TC, Mulrow CD, et al. The PRISMA 2020 statement: an updated guideline for reporting systematic reviews. BMJ. 2021;372:n71. 10.1136/bmj.n71. 10.1136/bmj.n71 PMC8005924 33782057

[16] Garcia-Rea EA, LePage JP. Effects of childhood abuse on relapse in a recently homeless substance-dependent veteran population. Psychological Trauma: Theory, Res, Pract, Policy. 2010;2(3):201–05. 10.1037/a0019186.

[17] Godley MD, Godley SH, Dennis ML, Funk RR, Passetti LL. The effect of assertive continuing care on continuing care linkage, adherence and abstinence following residential treatment for adolescents with substance use disorders. Addiction. 2006;102(1):81–93. 10.1111/j.1360-0443.2006.01648.x. 10.1111/j.1360-0443.2006.01648.x 17207126

[18] Goldstein RB, Bigelow C, McCusker J, Lewis BF, Mundt KA, Powers SI. Antisocial behavioral syndromes and return to drug use following residential relapse prevention/health education treatment. Am J Drug Alcohol Abuse. 2001;27(3):453–82. 10.1081/ADA-100104512. 11506262 10.1081/ada-100104512

[19] González-Roz A, Castaño Y, Krotter A, Salazar-Cedillo A, Gervilla E. Emotional dysregulation in relation to substance use and behavioral addictions: findings from five separate meta-analyses. Int J Clin Health Phychol. 2024;24(3):100502. 10.1016/j.ijchp.2024.100502. 10.1016/j.ijchp.2024.100502 PMC11416520 39308782

[20] Graham L, Das J, Vitorio R, McDonald C, Walker R, Godfrey A, et al. Ocular microtremor: a structured review. Exp Brain Res. 2023;241(9):2191–203. 10.1007/s00221-023-06691-w. 37632535 10.1007/s00221-023-06691-w PMC10471653

[21] Gray C, Argaez C. Residential treatment for substance use disorder: a review of clinical effectiveness. Canadian Agency for Drugs and Technologies in Health; 2019. https://www.ncbi.nlm.nih.gov/books/NBK541232/. 31107598

[22] Groh D, Jason LA, Ferrari J, Halpert J. A longitudinal investigation of the predictability of the three-factor model of the important people Inventory. Am J Drug Alcohol Abuse. 2011;37(4):259–63. 10.3109/00952990.2011.591017. 21702726 10.3109/00952990.2011.591017 PMC3582215

[23] Gurnani B, Kaur K. Modified drug regimen for ophthalmic anesthesia during COVID-19 pandemic: revisiting pharmacological concepts. Indian J Pharmacol. 2021;53(2):178–79. 10.4103/ijp.ijp_89_21. 34100406 10.4103/ijp.ijp_89_21 PMC8265423

[24] Gustafson DH, McTavish FM, Chih M-Y, Atwood AK, Johnson RA, Boyle MG, et al. A smartphone application to support recovery from alcoholism. JAMA Psychiatry. 2014;71(5):566. 10.1001/jamapsychiatry.2013.4642. 24671165 10.1001/jamapsychiatry.2013.4642 PMC4016167

[25] Hajihosseini M, Norozi E, Azimi T, Ahmadinia H. Comparison of artificial neural networks and logistic regression for determination of social-cognitive factors impacting drug abstinence. J Subst Use. 2019;25(3):295–300. 10.1080/14659891.2019.1690060.

[26] Higgins J, Thomas J. Cochrane handbook for systematic reviews of interventions. Cochrane Training; 2023. https://training.cochrane.org/handbook/current.

[27] Jainchill N, Hawke J, de Leon G, Yagelka J. Adolescents in therapeutic communities: one-year posttreatment outcomes. J Psychoact Drugs. 2000;32(1):81–94. 10.1080/02791072.2000.10400214. 10.1080/02791072.2000.10400214 10801070

[28] Januszko P, Gmaj B, Piotrowski T, Kopera M, Klimkiewicz A, Wnorowska A, et al. Delta resting-state functional connectivity in the cognitive control network as a prognostic factor for maintaining abstinence: an eLORETA preliminary study. Drug Alcohol Depen. 2021;218:108393. 10.1016/j.drugalcdep.2020.108393. 10.1016/j.drugalcdep.2020.108393 33158664

[29] Jean YK, Kam D, Gayer S, Palte HD, Stein ALS. Regional anesthesia for pediatric ophthalmic surgery: a review of the literature. Anesth Analg. 2020;130(5):1351–63. 10.1213/ANE.0000000000004012. 30676353 10.1213/ANE.0000000000004012

[30] Johnston AL, Thevos AK, Randall CL, Anton RF. Increased severity of alcohol withdrawal in in-patient alcoholics with a co-existing anxiety diagnosis. Br J Addict. 1991;86(6):719–25. 10.1111/j.1360-0443.1991.tb03098.x. 1878622 10.1111/j.1360-0443.1991.tb03098.x

[31] Kabisa E, Biracyaza E, Habagusenga JD, Umubyeyi A. Determinants and prevalence of relapse among patients with substance use disorders: case of icyizere psychotherapeutic centre. Subst Abuse Treat Prev Policy. 2021;16(1):1–12. 10.1186/s13011-021-00347-0. 33526066 10.1186/s13011-021-00347-0 PMC7851925

[32] Keyser-Marcus L, Alvanzo A, Rieckmann T, Thacker L, Sepulveda A, Forcehimes A, et al. Trauma, gender, and mental health symptoms in individuals with substance use disorders. J Interpers Violence. 2015;30(1):3–24. 10.1177/0886260514532523. 24811286 10.1177/0886260514532523 PMC4766974

[33] Klein AA, Slaymaker VJ. 12-step involvement and treatment outcomes among young women with substance use disorders. Alcohol Treat Q. 2011;29(3):204–18. 10.1080/07347324.2011.586288.

[34] Klein AA, Slaymaker VJ, Dugosh KL, McKay JR. Computerized continuing care support for alcohol and drug dependence: a preliminary analysis of usage and outcomes. J Subst Abuse Treat. 2012;42(1):25–34. 10.1016/j.jsat.2011.07.002. 21862275 10.1016/j.jsat.2011.07.002 PMC6498848

[35] Kolla BP, Schneekloth T, Mansukhani MP, Biernacka JM, Hall-Flavin D, Karpyak V, et al. The association between sleep disturbances and alcohol relapse: a 12-month observational cohort study. Am J Addict. 2015;24(4):362–67. 10.1111/ajad.12199. 25808396 10.1111/ajad.12199

[36] Laffaye C, McKellar JD, Ilgen MA, Moos RH. Predictors of 4-year outcome of community residential treatment for patients with substance use disorders. Addiction. 2008;103(4):671–80. 10.1111/j.1360-0443.2008.02147.x. 18339113 10.1111/j.1360-0443.2008.02147.x

[37] Lin L, Chu H. Quantifying publication bias in meta-analysis. Biometrics. 2017;74(3):785–94. 10.1111/biom.12817. 29141096 10.1111/biom.12817 PMC5953768

[38] Ludwig F, Tadayon-Manssuri E, Strik W, Moggi F. Self-efficacy as a predictor of outcome after residential treatment programs for alcohol dependence: simply ask the patient one question! Alcohol Clin Exp Res. 2013;37(4):663–67. 10.1111/acer.12007. 23075331 10.1111/acer.12007

[39] Majer JM, Beasley C, Stecker E, Bobak TJ, Norris J, Nguyen HM, et al. Oxford House residents’ attitudes toward medication assisted treatment use in fellow residents. Community Ment Health J. 2018;54(5):571–77. 10.1007/s10597-017-0218-4. 29302767 10.1007/s10597-017-0218-4

[40] Mandell W, Edelen MO, Wenzel SL, Dahl J, Ebener P. Do dimensions of therapeutic community treatment predict retention and outcomes? J Subst Abuse Treat. 2008;35(3):223–31. 10.1016/j.jsat.2007.10.004. 18248942 10.1016/j.jsat.2007.10.004 PMC3623973

[41] McKellar JD, Harris AH, Moos RH. Patients’ abstinence status affects the benefits of 12-step self-help group participation on substance use disorder outcomes. Drug Alcohol Depen. 2009;99(1–3):115–22. 10.1016/j.drugalcdep.2008.07.005. 10.1016/j.drugalcdep.2008.07.005 18778901

[42] McKetin R, Kothe A, Baker AL, Lee NK, Ross J, Lubman DI. Predicting abstinence from methamphetamine use after residential rehabilitation: findings from the methamphetamine treatment evaluation study. Drug Alcohol Rev. 2018;37(1):70–78. 10.1111/dar.12528. 28421682 10.1111/dar.12528

[43] Mericle AA, Mahoney E, Korcha R, Delucchi K, Polcin DL. Sober living house characteristics: a multilevel analyses of factors associated with improved outcomes. J Subst Abuse Treat. 2019;98:28–38. 10.1016/j.jsat.2018.12.004. 30665601 10.1016/j.jsat.2018.12.004 PMC6605057

[44] Michaud-Germain C, Tardif P-A, Nadeau A, Gagnon A-P, Mercier É. Contribution of substance use in acute injuries with regards to the intent, nature and context of injury: a CHIRPP database study. Cureus. 2020. 10.7759/cureus.10282. 10.7759/cureus.10282 PMC7538210 33042717

[45] Mikhail M, Sabri K, Levin AV. Effect of anesthesia on intraocular pressure measurement in children. Surv Ophthalmol. 2017;62(5):648–58. 10.1016/j.survophthal.2017.04.003. 28438591 10.1016/j.survophthal.2017.04.003

[46] Miller D, Lewis SR, Pritchard MW, Schofield-Robinson OJ, Shelton CL, Alderson P, et al. Intravenous versus inhalational maintenance of anaesthesia for postoperative cognitive outcomes in elderly people undergoing non-cardiac surgery. Cochrane Database Systematic Rev. 2018;2018(10):CD012317. 10.1002/14651858.CD012317.pub2. 10.1002/14651858.CD012317.pub2 PMC6513211 30129968

[47] Mo C, Deane FP. Reductions in craving and negative affect predict 3-month post-discharge alcohol use following residential treatment. Int J Ment Health Addict. 2016;14(5):761–74. 10.1007/s11469-015-9626-2.

[48] Mohankumar A, Rajan M. Role of hyaluronidase as an adjuvant in local anesthesia for cataract surgery. Indian J Ophthalmol. 2023;71(7):2649–55. 10.4103/ijo.IJO_2515_22. 37417102 10.4103/IJO.IJO_2515_22 PMC10491076

[49] Mulhauser K, Weinstock J, Van Patten R, McGrath AB, Merz ZC, White CN. Examining the stability of the UPPS-P and MCQ-27 during residential treatment for substance use disorder. Exp Clin Psychopharmacol. 2019;27(5):474–81. 10.1037/pha0000255. 30628814 10.1037/pha0000255

[50] Polcin DL, Henderson DM. A clean and sober place to live: philosophy, structure, and purported therapeutic factors in sober living houses. J Psychoact Drugs. 2008;40(2):153–59. 10.1080/02791072.2008.10400625. 10.1080/02791072.2008.10400625 PMC2556949 18720664

[51] Polcin DL, Korcha RA, Bond J, Galloway G. Sober living houses for alcohol and drug dependence: 18-month outcomes. J Subst Abuse Treat. 2010;38(4):356–65. 10.1016/j.jsat.2010.02.003. 20299175 10.1016/j.jsat.2010.02.003 PMC2860009

[52] Polcin DL, Korcha R. Housing status, psychiatric symptoms, and substance abuse outcomes among sober living house residents over 18 months. Addictive Disord Their Treat. 2017;16(3):138–50. 10.1097/ADT.0000000000000105. 10.1097/ADT.0000000000000105 PMC5646694 29056875

[53] Ponzer S, Johansson S-E, Bergman B. A four-year follow-up study of male alcoholics: factors affecting the risk of readmission. Alcohol. 2002;27(2):83–88. 10.1016/S0741-8329(02)00203-3. 12106827 10.1016/s0741-8329(02)00203-3

[54] Reif S, George P, Braude L, Dougherty RH, Daniels AS, Ghose SS, et al. Residential treatment for individuals with substance use disorders: assessing the evidence. Psychiatric Serv. 2014;65(3):301–12. 10.1176/appi.ps.201300242. 10.1176/appi.ps.201300242 24445598

[55] Rifai MA, Moles JK, Lehman LP, Van der Linden BJ. Hepatitis C screening and treatment outcomes in patients with substance use/dependence disorders. Psychosomatics. 2006;47(2):112–21. 10.1176/appi.psy.47.2.112. 16508022 10.1176/appi.psy.47.2.112

[56] Rollins AL, O’Neill SJ, Davis KE, Devitt TS. Special Section on relapse prevention: substance Abuse relapse and factors associated with relapse in an inner-city sample of patients with dual diagnoses. Psychiatric Serv. 2005;56(10):1274–81. 10.1176/appi.ps.56.10.1274. 10.1176/appi.ps.56.10.1274 16215195

[57] Sannibale C, Hurkett P, Van den Bossche E, O’Connor D, Zador D, Capus C, et al. Aftercare attendance and post-treatment functioning of severely substance dependent residential treatment clients. Drug Alcohol Rev. 2003;22(2):181–90. 10.1080/09595230100100624. 12850905 10.1080/09595230100100624

[58] Sinclair DL, Sussman S, De Schryver M, Samyn C, Adams S, Florence M, et al. Substitute behaviors following residential substance use treatment in the Western Cape, South Africa. IJERPH. 2021;18(23):12815. 10.3390/ijerph182312815. 34886541 10.3390/ijerph182312815 PMC8657596

[59] United Nations Office on Drugs and Crime. World drug report 2023. 2023. https://www.unodc.org/res/WDR-2023/WDR23_Exsum_fin_DP.pdf.

[60] van Hagen LJ, de Waal MM, Christ C, Dekker JJM, Goudriaan AE. Patient characteristics predicting abstinence in substance use disorder patients with comorbid mental disorders. J Dual Diagnos. 2019;15(4):312–23. 10.1080/15504263.2019.1667043. 10.1080/15504263.2019.1667043 31566115

[61] Weiss NH, Kiefer R, Goncharenko S, Raudales AM, Forkus SR, Schick MR, et al. Emotion regulation and substance use: a meta-analysis. Drug Alcohol Depen. 2021;230:109131. 10.1016/j.drugalcdep.2021.109131. 10.1016/j.drugalcdep.2021.109131 PMC8714680 34864568

[62] Ober AJ, Osilla KC, Klein DJ, Burgette LF, Leamon I, Mazer MW, et al. Pilot randomized controlled trial of a hospital-based substance use treatment and recovery team (START) to improve initiation of medication for alcohol or opioid use disorder and linkage to follow-up care. J Subst Use Addict Treat. 2023;150:209063. 10.1016/j.josat.2023.209063. 37156424 10.1016/j.josat.2023.209063 PMC10330512

[63] Kelly PJ, Ingram I, Deane FP, Baker AL, Byrne G, Degan T, et al. Feasibility and preliminary results of a call centre delivered continuing care intervention following residential alcohol and other drug treatment. Drug Alcohol Rev. 2023;42(6):1395–405. 10.1111/dar.13693. 37248675 10.1111/dar.13693

[64] Watson DP, Tillson M, Taylor L, Xu H, Ouyang F, Beaudoin FL, et al. Results from the POINT pragmatic randomized trial: an emergency department-based peer support specialist intervention to increase opioid use disorder treatment linkage and reduce recurrent overdose. Subst Use Addict J. 2024;45(3):378–89. 10.1177/29767342231221054. 10.1177/29767342231221054 PMC11179981 38258819

[65] Lang C, Weisel KK, Saur S, Fuhrmann LM, Schoenleber A, Reichl D, et al. Support after return to alcohol use: a mixed-methods study on how abstinence motivation and app use change after return to alcohol use in an app-based aftercare intervention for individuals with alcohol use disorder. Addict Sci Clin Pract. 2024;19(1):35. 10.1186/s13722-024-00457-7. 38711152 10.1186/s13722-024-00457-7 PMC11071226

[66] Kelly PJ, Deane FP, Baker AL, Townsend C, McKay JR, Degan TJ, et al. The continuing care project: a multi-arm randomised controlled trial of a continuing care telephone intervention following residential substance use treatment. Drug Alcohol Depen. 2025;272:112668. 10.1016/j.drugalcdep.2025.112668. 10.1016/j.drugalcdep.2025.112668 40288058

[67] Saur S, Weisel KK, Lang C, Fuhrmann LM, Meurer N, Reichl D, et al. Evaluation of a smartphone app intervention with telephone guidance as transitional support from inpatient treatment to continuing care for individuals with alcohol use disorder: results from a randomized controlled trial. Psychother Psychosom. 2025;94(5):311–23. 10.1159/000545817. 40414214 10.1159/000545817

[68] Garland EL, Franken IHA, Howard MO. Cue-elicited heart rate variability and attentional bias predict alcohol relapse following treatment. Psychopharmacology. 2012;222(1):17–26. 10.1007/s00213-011-2618-4. 22203318 10.1007/s00213-011-2618-4 PMC3346884

[69] Suter M, Strik W, Moggi F. Depressive symptoms as a predictor of alcohol relapse after residential treatment programs for alcohol use disorder. J Subst Abuse Treat. 2011;41(3):225–32. 10.1016/j.jsat.2011.03.005. 21546204 10.1016/j.jsat.2011.03.005

[70] Chou JL, Cooper-Sadlo S, Diamond RM, Muruthi BA, Beeler-Stinn S. An exploration of mothers’ successful completion of family-centered residential substance use treatment. Fam Process. 2020;59(3):1113–27. 10.1111/famp.12501. 31617203 10.1111/famp.12501 PMC7160039

[71] Chou JL, Noel JG, Williams C, Spruell S, Nixon K, Riedel E, et al. Residential substance use treatment outcomes for pregnant and postpartum women: distinct patterns for women enrolled before versus during the COVID-19 pandemic. J Nurs Scholarsh. 2023;55(3):730–38. 10.1111/jnu.12803. 35920587 10.1111/jnu.12803 PMC9539050

[72] Vanderplasschen W, Colpaert K, Broekaert E. Determinants of relapse and re-admission among alcohol abusers after intensive residential treatment. Arch Public Health. 2010;67(4):194–211. 10.1186/0778-7367-67-4-194.

[73] Chong J. Predictors of relapse for American Indian women after substance abuse treatment. AIANMHR. 2008;14(3):24–48. 10.5820/aian.1403.2007.24. 10.5820/aian.1403.2007.24 18286445

[74] Dingwall KM, Maruff P, Cloughh AR, Cairney S. Factors associated with continued solvent use in Indigenous petrol sniffers following treatment. Drug Alcohol Rev. 2012;31(1):40–46. 10.1111/j.1465-3362.2010.00279.x. 21355931 10.1111/j.1465-3362.2010.00279.x

[75] Cavuoto KM, Javitt M, Chang TC. Neurodevelopmental effect of general anesthesia on the pediatric patient. J Pediatr Ophthalmol Strabismus. 2019;56(6):349–53. 10.3928/01913913-20190923-03. 31743401 10.3928/01913913-20190923-03

[76] de Andrade D, Elphinston RA, Quinn C, Allan J, Hides L. The effectiveness of residential treatment services for individuals with substance use disorders: a systematic review. Drug Alcohol Depen. 2019;201:227–35. 10.1016/j.drugalcdep.2019.03.031. 10.1016/j.drugalcdep.2019.03.031 31254749

[77] Decker KP, Peglow SL, Samples CR, Cunningham TD. Long-term outcomes after residential substance use treatment: relapse, morbidity, and mortality. Mil Med. 2017;182(1):e1589–95. 10.7205/MILMED-D-15-00560. 10.7205/MILMED-D-15-00560 28051978

[78] Dhillon HK, Agarkar S, Vijaya L, Bhende M, Baskaran M, Jaichandran VV. Examination under anesthesia: preferred practice. Indian J Ophthalmol. 2023;71(11):3438–45. 10.4103/ijo.IJO_3259_22. 37870002 10.4103/IJO.IJO_3259_22 PMC10752321

[79] Glass JE, McKay JR, Gustafson DH, Kornfield R, Rathouz PJ, McTavish FM, et al. Treatment seeking as a mechanism of change in a randomized controlled trial of a mobile health intervention to support recovery from alcohol use disorders. J Subst Abuse Treat. 2017;77:57–66. 10.1016/j.jsat.2017.03.011. 28476273 10.1016/j.jsat.2017.03.011 PMC5880274

[80] Gossop M, Marsden J, Stewart D, Rolfe A. Treatment retention and 1 year outcomes for residential programmes in England. Drug Alcohol Depen. 1999;57(2):89–98. 10.1016/S0376-8716(99)00086-1. 10.1016/s0376-8716(99)00086-1 10617094

[81] Gupta Y, Shanmugam C, K P, Mandal S, Tandon R, Sharma N. Pediatric keratoconus. Surv Ophthalmol. 2025;70(2):296–330. 10.1016/j.survophthal.2024.10.003. 39396644 10.1016/j.survophthal.2024.10.003

[82] McHugh TA. Implications of inspired carbon dioxide during ophthalmic surgery performed using monitored anesthesia care. Aana J. 2019;87(4):285–90. 31587712

[83] Mutschler C, Junaid S, Tellez C, Franco G, Gryspeerdt C, Bushe J. Community-based residential treatment for alcohol and substance use problems: a realist review. Health Soc Care Comm. 2021;30(2). 10.1111/hsc.13511. 10.1111/hsc.13511 35122344

[84] Proctor SL, Wainwright JL, Herschman PL. Importance of short-term continuing care plan adherence on long-term outcomes among patients discharged from residential substance use treatment. Am J Drug Alcohol Abuse. 2017;43(6):734–41. 10.1080/00952990.2017.1329315. 28557531 10.1080/00952990.2017.1329315

[85] Statista Research Department. Topic: global drug use. 2023 August 31. https://www.statista.com/topics/7786/global-drug-use/#topicOverview. Statista.

[86] Curran GM, Flynn HA, Kirchner J, Booth BM. Depression after alcohol treatment as a risk factor for relapse among male veterans. J Subst Abuse Treat. 2000;19(3):259–65. 10.1016/S0740-5472(00)00107-0. 11027896 10.1016/s0740-5472(00)00107-0

[87] Driessen M. The course of anxiety, depression and drinking behaviours after completed detoxification in alcoholics with and without comorbid anxiety and depressive disorders. Alcohol Alcohol. 2001;36(3):249–55. 10.1093/alcalc/36.3.249. 11373263 10.1093/alcalc/36.3.249

[88] Eslami AA, Norozi E, Hajihosseini M, Ramazani AA, Miri MR. Social cognitive theory as a theoretical framework to predict sustained abstinence 6 months after substance use treatment. J Subst Use. 2018;23(3):300–06. 10.1080/14659891.2017.1394382.

[89] Müller A, Znoj H, Moggi F. How are self-efficacy and motivation related to drinking five years after residential treatment a longitudinal multicenter study. Eur Addict Res. 2019;25(5):213–23. 10.1159/000500520. 31112984 10.1159/000500520

[90] Bruce SE, Yonkers KA, Otto MW, Eisen JL, Weisberg RB, Pagano M, et al. Influence of psychiatric comorbidity on recovery and recurrence in generalized anxiety disorder, social phobia, and panic disorder: a 12-year prospective study. AJP. 2005;162(6):1179–87. 10.1176/appi.ajp.162.6.1179. 10.1176/appi.ajp.162.6.1179 PMC3272761 15930067

[91] Chermack ST, Murray RL, Walton MA, Booth BA, Wryobeck J, Blow FC. Partner aggression among men and women in substance use disorder treatment: correlates of psychological and physical aggression and injury. Drug Alcohol Depen. 2008;98(1–2):35–44. 10.1016/j.drugalcdep.2008.04.010. 10.1016/j.drugalcdep.2008.04.010 PMC3771635 18554825

[92] Shrier LA, Harris SK, Kurland M, Knight JR. Substance use problems and associated psychiatric symptoms among adolescents in primary care. Pediatrics. 2003;111(6):e699–705. 10.1542/peds.111.6.e699. 10.1542/peds.111.6.e699 12777588

[93] Stellern J, Xiao KB, Grennell E, Sanches M, Gowin JL, Sloan ME. Emotion regulation in substance use disorders: a systematic review and meta-analysis. Addiction. 2022;118(1):30–47. 10.1111/add.16001. 35851975 10.1111/add.16001 PMC10087816

[94] Flora K. A review of the factors affecting the course and outcome of the treatment of substance use disorders. J Subst Use. 2018;24(2):120–24. 10.1080/14659891.2018.1549598.

[95] Mutter R, Ali MM, Smith K, Strashny A. Factors associated with substance use treatment completion in residential facilities. Drug Alcohol Depen. 2015;154:291–95. 10.1016/j.drugalcdep.2015.07.004. 10.1016/j.drugalcdep.2015.07.004 26210736

[96] Collazzoni A, Stratta P, Pacitti F, Rossi A, Santarelli V, Bustini M, et al. Resilience as a mediator between interpersonal risk factors and hopelessness in depression. Front Psychiatry. 2020;11:10. 10.3389/fpsyt.2020.00010. 32184740 10.3389/fpsyt.2020.00010 PMC7059212

[97] Brown TG, Seraganian P, Tremblay J, Annis H. Process and outcome changes with relapse prevention versus 12-step aftercare programs for substance abusers. Addiction. 2002;97(6):677–89. 10.1046/j.1360-0443.2002.00101.x. 12084137 10.1046/j.1360-0443.2002.00101.x

[98] Kornfield R, Toma CL, Shah DV, Moon TJ, Gustafson DH. What do you say before you relapse? How language use in a peer-to-peer online discussion forum predicts risky drinking among those in recovery. Health Commun. 2018;33(9):1184–93. 10.1080/10410236.2017.1350906. 28792228 10.1080/10410236.2017.1350906 PMC6059378

[99] Lenzi L, Ibrahim A, Brough D, Thompson A. Digital interventions for supporting alcohol abstinence in aftercare – a systematic review. Internet Interventions. 2025;40:100832. 10.1016/j.invent.2025.100832. 40486131 10.1016/j.invent.2025.100832 PMC12143608

[100] Substance Abuse and Mental Health Services Administration (US). Early intervention, treatment, and management of substance use disorders. US. Department of Health and Human Services; 2019 November. https://www.ncbi.nlm.nih.gov/books/NBK424859/.

[101] Groh DR, Jason LA, Davis MI, Olson BD, Ferrari JR. Friends, family, and alcohol abuse: an examination of general and alcohol-specific social support. Am J Addict. 2007;16(1):49–55. 10.1080/10550490601080084. 17364422 10.1080/10550490601080084 PMC2977912

[102] National Institute of Health. Study quality assessment tools | national heart, lung, and Blood institute (NHLBI). 2021. https://www.nhlbi.nih.gov/health-topics/study-quality-assessment-tools.

[103] Chen J, Zou X, Hu B, Yang Y, Wang F, Zhou Q, et al. Remimazolam vs etomidate: haemodynamic effects in hypertensive elderly patients undergoing non-cardiac surgery. DDDT. 2023;17:2943–53. 10.2147/DDDT.S425590. 37789968 10.2147/DDDT.S425590 PMC10544010

[104] Chua AW, Kumar CM, Harrisberg BP, Eke T. Anaesthetic considerations for the surgical management of ocular hypotony in adults. Anaesth Intensive Care. 2023;51(2):107–13. 10.1177/0310057X221111183. 36524304 10.1177/0310057X221111183

[105] De Nucci A, Scialdone A, Lando G, Monaco G, Cacioppo V, Campbell Davies S, et al. Effectiveness and safety of intravenous dexmedetomidine sedation for ophthalmic surgery under regional anesthesia. Eur J Ophthalmol. 2022;32(5):2598–603. 10.1177/11206721211059013. 34766512 10.1177/11206721211059013

[106] Mantagos IS, Kleinman ME, Kieran MW, Gordon LB. Ophthalmologic features of progeria. Am J Ophthalmol. 2017;182:126–32. 10.1016/j.ajo.2017.07.020. 28756152 10.1016/j.ajo.2017.07.020

[107] Li MY, Fei YD, Zhang XX, Chen TW, Li J, Sun XL, et al. Application of propofol-remifentanil intravenous general anesthesia combined with regional block in pediatric ophthalmic surgery. BMC Anesthesiol. 2024;24(1):147. 10.1186/s12871-024-02531-8. 38632505 10.1186/s12871-024-02531-8 PMC11022467

[108] Darke S, Williamson A, Ross J, Teesson M. Residential rehabilitation for the treatment of heroin dependence: sustained heroin abstinence and drug-related problems 2 years after treatment entrance. Addictive Disord Their Treat. 2006;5(1):9–18. 10.1097/01.ADT.0000210702.63165.81.

[109] Savage C. Residential psychedelic (LSD) therapy for the narcotic addict. Arch Gen Psychiatry. 1973;28(6):808. 10.1001/archpsyc.1973.01750360040005. 4575166 10.1001/archpsyc.1973.01750360040005

[110] Hser YI, Joshi V, Anglin MD, Fletcher B. Predicting posttreatment cocaine abstinence for first-time admissions and treatment repeaters. Am J Public Health. 1999;89(5):666–71. 10.2105/AJPH.89.5.666. 10224976 10.2105/ajph.89.5.666 PMC1508725

[111] Jayarajan AP, Sharma A, Sharma R, Nirankari VS, Narayana S, Christy JS. Congenital corneal anesthesia: a case series. Indian J Ophthalmol. 2022;70(7):2588–91. 10.4103/ijo.IJO_3001_21. 35791163 10.4103/ijo.IJO_3001_21 PMC9426152

[112] Miller JM, Miller HV. Considering the effectiveness of drug treatment behind bars: findings from the South Carolina RSAT evaluation. Justice Q. 2011;28(1):70–86. 10.1080/07418825.2010.506880.

[113] Welsh WN. A multisite evaluation of prison-based therapeutic community drug treatment. Crim Justice Behav. 2007;34(11):1481–98. 10.1177/0093854807307036.

