# Supplementary material for: Risk and protective factors for relapse after residential substance use treatment: a systematic review and meta-analysis
Source: BMC Psychiatry. 2026 Jul 23;26:676. doi: 10.1186/s12888-026-08330-4 (PMC13520516; doi:10.1186/s12888-026-08330-4)
Supplement: Supplementary file 1 [file 12888_2026_8330_MOESM1_ESM.docx]

**Supplementary Appendix S1. Full Search Strategies and Grey Literature Sources**

**S1.0 Last search date**

**Last search date (all sources): 31 October 2023**
**Language:** English
**Population:** Humans (where database filter available)
**Study design:** No design filters applied at search stage (screened during selection)

**S1.1 Database search strategies (exact strings)**

| **Database** | **Platform** | **Date searched** | **Search string (exact)** | **Limits/filters applied** |
| --- | --- | --- | --- | --- |
| PubMed | NCBI | 31 Oct 2023 | ((relapse[Title/Abstract] OR recidivism[Title/Abstract] OR recurrence[Title/Abstract] OR "return to use"[Title/Abstract] OR "return to drinking"[Title/Abstract] OR "return to drug use"[Title/Abstract] OR abstinen*[Title/Abstract] OR remission[Title/Abstract] OR readmission[Title/Abstract] OR "treatment readmission"[Title/Abstract] OR "treatment re-entry"[Title/Abstract] OR "treatment reentry"[Title/Abstract] OR "continued use"[Title/Abstract] OR "lapse"[Title/Abstract]) AND (residential[Title/Abstract] OR "therapeutic community"[Title/Abstract] OR inpatient[Title/Abstract] OR "in-patient"[Title/Abstract] OR "licensed facility"[Title/Abstract] OR detox[Title/Abstract] OR "detoxification"[Title/Abstract] OR "24-hour"[Title/Abstract] OR "24 hour"[Title/Abstract] OR "rehabilitation facility"[Title/Abstract] OR "rehab"[Title/Abstract]) AND ("substance use disorder"[Title/Abstract] OR "substance-related disorder"[Title/Abstract] OR "substance abuse"[Title/Abstract] OR "substance dependence"[Title/Abstract] OR "drug use disorder"[Title/Abstract] OR alcohol[Title/Abstract] OR opioid*[Title/Abstract] OR heroin[Title/Abstract] OR cocaine[Title/Abstract] OR methamphetamine[Title/Abstract] OR stimulant*[Title/Abstract] OR cannabis[Title/Abstract] OR "drug abuse"[Title/Abstract] OR "drug dependence"[Title/Abstract])) | English; Humans (PubMed filter); no date restriction |
| Embase | Elsevier Embase.com | 31 Oct 2023 | ('relapse'/exp OR relapse:ti,ab OR recidivism:ti,ab OR recurrence:ti,ab OR 'return to use':ti,ab OR 'return to drinking':ti,ab OR 'return to drug use':ti,ab OR abstinen*:ti,ab OR remission:ti,ab OR readmission:ti,ab OR 'treatment readmission':ti,ab OR 'treatment re-entry':ti,ab OR lapse:ti,ab) AND ('residential treatment'/exp OR residential:ti,ab OR 'therapeutic community':ti,ab OR inpatient:ti,ab OR 'licensed facility':ti,ab OR detox:ti,ab OR detoxification:ti,ab OR '24-hour':ti,ab OR 'rehabilitation facility':ti,ab OR rehab:ti,ab) AND ('substance use disorder'/exp OR 'substance abuse'/exp OR 'drug dependence'/exp OR 'alcohol dependence'/exp OR 'opioid dependence'/exp OR 'substance use disorder':ti,ab OR alcohol:ti,ab OR opioid*:ti,ab OR heroin:ti,ab OR cocaine:ti,ab OR methamphetamine:ti,ab OR stimulant*:ti,ab OR cannabis:ti,ab) | English; Humans; no date restriction |
| Scopus | Elsevier Scopus | 31 Oct 2023 | TITLE-ABS-KEY ( relapse OR recidivism OR recurrence OR "return to use" OR "return to drinking" OR "return to drug use" OR abstinen* OR remission OR readmission OR "treatment readmission" OR "treatment re-entry" OR lapse ) AND TITLE-ABS-KEY ( residential OR "therapeutic community" OR inpatient OR "licensed facility" OR detox OR detoxification OR "24-hour" OR "rehabilitation facility" OR rehab ) AND TITLE-ABS-KEY ( "substance use disorder" OR "substance abuse" OR "substance dependence" OR "drug use disorder" OR "drug dependence" OR alcohol OR opioid* OR heroin OR cocaine OR methamphetamine OR stimulant* OR cannabis ) | English; no date restriction |
| PsycINFO | APA PsycINFO (via ProQuest) | 31 Oct 2023 | (TI,AB(relapse OR recidivism OR recurrence OR "return to use" OR "return to drinking" OR "return to drug use" OR abstinen* OR remission OR readmission OR lapse) ) AND (TI,AB(residential OR "therapeutic community" OR inpatient OR detox OR detoxification OR "rehabilitation facility" OR rehab OR "24-hour") ) AND (TI,AB("substance use disorder" OR "substance abuse" OR "substance dependence" OR "drug use disorder" OR alcohol OR opioid* OR heroin OR cocaine OR methamphetamine OR stimulant* OR cannabis) ) | English; no date restriction |
| Web of Science Core Collection | Clarivate Web of Science | 31 Oct 2023 | TS=(relapse OR recidivism OR recurrence OR "return to use" OR "return to drinking" OR "return to drug use" OR abstinen* OR remission OR readmission OR "treatment readmission" OR "treatment re-entry" OR lapse) AND TS=(residential OR "therapeutic community" OR inpatient OR detox OR detoxification OR "24-hour" OR "rehabilitation facility" OR rehab OR "licensed facility") AND TS=("substance use disorder" OR "substance abuse" OR "substance dependence" OR "drug use disorder" OR alcohol OR opioid* OR heroin OR cocaine OR methamphetamine OR stimulant* OR cannabis) | English; no date restriction |

**S1.2 Grey literature search (sources and approach)**

| **Grey literature source** | **Platform** | **Date searched** | **Query/search approach (exact)** | **Notes/limits** |
| --- | --- | --- | --- | --- |
| ProQuest Dissertations & Theses Global | ProQuest | 31 Oct 2023 | ("residential treatment" OR "therapeutic community" OR inpatient OR detox OR detoxification) AND (relapse OR abstinen* OR readmission OR "return to use") AND ("substance use disorder" OR alcohol OR opioid* OR heroin OR cocaine OR methamphetamine) | English; screened titles/abstracts then full text where available |
| Google Scholar | Web | 31 Oct 2023 | allintitle: (relapse OR abstinence OR readmission) (residential OR "therapeutic community") ("substance use disorder" OR alcohol OR opioid OR heroin OR cocaine OR methamphetamine) | First 200 results screened (sorted by relevance); duplicates removed |
| Conference proceedings | Web of Science/Scopus (conference filters where available) | 31 Oct 2023 | Used the same core strategy as database search; filtered to conference/proceedings where available | Screened as potential grey literature |
| Manual web searching of organizational/agency reports | Web | 31 Oct 2023 | Search within relevant organizational sites using terms: relapse, residential treatment, aftercare, substance use disorder | Included only if primary data and eligible design |
| Reference list screening | Manual | 31 Oct 2023 | Reference lists of included studies and relevant reviews were screened for additional eligible studies | Backward searching |
| “Related articles” / citation chasing | Platform tools | 31 Oct 2023 | PubMed “Similar articles” and citation links in Scopus/WoS used for forward/backward citation chasing | Applied eligibility screening |

**References**

1. Aas, C. F., Vold, J. H., Gjestad, R., Skurtveit, S., Lim, A. G., Gjerde, K. V., Løberg, E.-M., Johansson, K. A., & Fadnes, L. T. (2021). Substance use and symptoms of mental health disorders: A prospective cohort of patients with severe substance use disorders in Norway. Substance Abuse Treatment, Prevention, and Policy, 16(1). <https://doi.org/10.1186/s13011-021-00354-1>
2. Anand, D., Chen, Y., Lindquist, K. A., & Daughters, S. B. (2017). Emotion differentiation predicts likelihood of initial lapse following substance use treatment. Drug and Alcohol Dependence, 180, 439–444. <https://doi.org/10.1016/j.drugalcdep.2017.09.007>
3. Andersson, H. W., Lauvsnes, A. D. F., & Nordfjærn, T. (2021). Emerging adults in inpatient substance use treatment: A prospective cohort study of patient characteristics and treatment outcomes. European Addiction Research, 27(3), 206–215. <https://doi.org/10.1159/000512156>
4. Barreno, E. M., Domínguez-Salas, S., Díaz-Batanero, C., Lozano, Ó. M., Marín, J. A. L., & Verdejo-García, A. (2018). Specific aspects of cognitive impulsivity are longitudinally associated with lower treatment retention and greater relapse in therapeutic community treatment. Journal of Substance Abuse Treatment, 96, 33–38. <https://doi.org/10.1016/j.jsat.2018.10.004>
5. Bauer, S., Strik, W., & Moggi, F. (2014). Motivation as a predictor of drinking outcomes after residential treatment programs for alcohol dependence. Journal of Addiction Medicine, 8(2), 137–142. <https://doi.org/10.1097/ADM.0000000000000013>
6. Bergman, B. G., Greene, M. C., Slaymaker, V., Hoeppner, B. B., & Kelly, J. F. (2014). Young adults with co-occurring disorders: Substance use disorder treatment response and outcomes. Journal of Substance Abuse Treatment, 46(4), 420–428. <https://doi.org/10.1016/j.jsat.2013.11.005>
7. Bergman, B. G., Hoeppner, B. B., Nelson, L. M., Slaymaker, V., & Kelly, J. F. (2015). The effects of continuing care on emerging adult outcomes following residential addiction treatment. Drug and Alcohol Dependence, 153, 207–214. <https://doi.org/10.1016/j.drugalcdep.2015.05.017>
8. Best, D., Manning, V., Allsop, S., & Lubman, D. I. (2020). Does the effectiveness of mutual aid depend on compatibility with treatment philosophies offered at residential rehabilitation services? Addictive Behaviors, 103, 106221. <https://doi.org/10.1016/j.addbeh.2019.106221>
9. Betancourt, C. A., Kitsantas, P., Goldberg, D. G., & Hawks, B. A. (2022). Substance use relapse among veterans at termination of treatment for substance use disorders. Military Medicine, 187(11–12), e1422–e1431. <https://doi.org/10.1093/milmed/usab280>
10. Blonigen, D. M., Macia, K. S., Smelson, D., & Timko, C. (2020). Criminal recidivism among justice-involved veterans following substance use disorder residential treatment. Addictive Behaviors, 106, 106357. <https://doi.org/10.1016/j.addbeh.2020.106357>
11. Bosso, R. A., Ribeiro, A., Basqueira, A. P., de Lima, M. T., dos Santos, J. P., Perrenoud, L. O., & Ribeiro, M. (2021). Recovery housing program for drug addicts: Work patterns, substance abuse, and housing situation after a 6-month follow-up. Addictive Disorders & Their Treatment, 20(4), 454–462. <https://doi.org/10.1097/ADT.0000000000000273>
12. Bradizza, C. M., Stasiewicz, P. R., & Paas, N. D. (2006). Relapse to alcohol and drug use among individuals diagnosed with co-occurring mental health and substance use disorders: A review. Clinical Psychology Review, 26(2), 162–178. <https://doi.org/10.1016/j.cpr.2005.11.005>
13. Brown, T. G., Seraganian, P., Tremblay, J., & Annis, H. (2002). Process and outcome changes with relapse prevention versus 12-step aftercare programs for substance abusers. Addiction, 97(6), 677–689. <https://doi.org/10.1046/j.1360-0443.2002.00101.x>
14. Bruce, S. E., Yonkers, K. A., Otto, M. W., Eisen, J. L., Weisberg, R. B., Pagano, M., Shea, M. T., & Keller, M. B. (2005). Influence of psychiatric comorbidity on recovery and recurrence in generalized anxiety disorder, social phobia, and panic disorder: A 12-year prospective study. American Journal of Psychiatry, 162(6), 1179–1187. <https://doi.org/10.1176/appi.ajp.162.6.1179>
15. Castaldelli-Maia, J. M., & Bhugra, D. (2022). Analysis of global prevalence of mental and substance use disorders within countries: Focus on sociodemographic characteristics and income levels. International Review of Psychiatry, 34(1), 6–15. <https://doi.org/10.1080/09540261.2022.2040450>
16. Cavuoto, K. M., Javitt, M., & Chang, T. C. (2019). Neurodevelopmental effect of general anesthesia on the pediatric patient. Journal of Pediatric Ophthalmology and Strabismus, 56(6), 349–353. <https://doi.org/10.3928/01913913-20190923-03>
17. Chen, J., Zou, X., Hu, B., Yang, Y., Wang, F., Zhou, Q., & Shen, M. (2023). Remimazolam vs etomidate: Haemodynamic effects in hypertensive elderly patients undergoing non-cardiac surgery. Drug Design, Development and Therapy, 17, 2943–2953. <https://doi.org/10.2147/DDDT.S425590>
18. Chermack, S. T., Murray, R. L., Walton, M. A., Booth, B. A., Wryobeck, J., & Blow, F. C. (2008). Partner aggression among men and women in substance use disorder treatment: Correlates of psychological and physical aggression and injury. Drug and Alcohol Dependence, 98(1–2), 35–44. <https://doi.org/10.1016/j.drugalcdep.2008.04.010>
19. Chong, J., & Lopez, D. (2008). Predictors of relapse for American Indian women after substance abuse treatment. American Indian and Alaska Native Mental Health Research: The Journal of the National Center, 14(3).
20. Chou, J. L., Cooper-Sadlo, S., Diamond, R. M., Muruthi, B. A., & Beeler-Stinn, S. (2020). An exploration of mothers’ successful completion of family-centered residential substance use treatment. Family Process, 59(3), 1113–1127. <https://doi.org/10.1111/famp.12501>
21. Chou, J. L., Noel, J. G., Williams, C., Spruell, S., Nixon, K., Riedel, E., & Zaarur, A. (2023). Residential substance use treatment outcomes for pregnant and postpartum women: Distinct patterns for women enrolled before versus during the COVID-19 pandemic. Journal of Nursing Scholarship, 55(3), 730–738. <https://doi.org/10.1111/jnu.12803>
22. Chua, A. W., Kumar, C. M., Harrisberg, B. P., & Eke, T. (2023). Anaesthetic considerations for the surgical management of ocular hypotony in adults. Anaesthesia and Intensive Care, 51(2), 107–113. <https://doi.org/10.1177/0310057X221111183>
23. Collazzoni, A., Stratta, P., Pacitti, F., Rossi, A., Santarelli, V., Bustini, M., Talevi, D., Socci, V., & Rossi, R. (2020). Resilience as a mediator between interpersonal risk factors and hopelessness in depression. Frontiers in Psychiatry, 11, 10. <https://doi.org/10.3389/fpsyt.2020.00010>
24. Curran, G. M., Flynn, H. A., Kirchner, J., & Booth, B. M. (2000). Depression after alcohol treatment as a risk factor for relapse among male veterans. Journal of Substance Abuse Treatment, 19(3), 259–265. <https://doi.org/10.1016/S0740-5472(00)00107-0>
25. Darke, S., Williamson, A., Ross, J., & Teesson, M. (2006). Residential rehabilitation for the treatment of heroin dependence: Sustained heroin abstinence and drug-related problems 2 years after treatment entrance. Addictive Disorders & Their Treatment, 5(1), 9–18. <https://doi.org/10.1097/01.ADT.0000210702.63165.81>
26. De Nucci, A., Scialdone, A., Lando, G., Monaco, G., Cacioppo, V., Campbell Davies, S., Casalino, G., & Gemma, M. (2022). Effectiveness and safety of intravenous dexmedetomidine sedation for ophthalmic surgery under regional anesthesia. European Journal of Ophthalmology, 32(5), 2598–2603. <https://doi.org/10.1177/11206721211059013>
27. de Andrade, D., Elphinston, R. A., Quinn, C., Allan, J., & Hides, L. (2019). The effectiveness of residential treatment services for individuals with substance use disorders: A systematic review. Drug and Alcohol Dependence, 201, 227–235. <https://doi.org/10.1016/j.drugalcdep.2019.03.031>
28. Decker, K. P., Peglow, S. L., Samples, C. R., & Cunningham, T. D. (2017). Long-term outcomes after residential substance use treatment: Relapse, morbidity, and mortality. Military Medicine, 182(1), e1589–e1595. <https://doi.org/10.7205/MILMED-D-15-00560>
29. Dhillon, H. K., Agarkar, S., Vijaya, L., Bhende, M., Baskaran, M., & Jaichandran, V. V. (2023). Examination under anesthesia: Preferred practice. Indian Journal of Ophthalmology, 71(11), 3438–3445. <https://doi.org/10.4103/ijo.IJO_3259_22>
30. Dingwall, K. M., Maruff, P., Cloughh, A. R., & Cairney, S. (2012). Factors associated with continued solvent use in Indigenous petrol sniffers following treatment. Drug and Alcohol Review, 31(1), 40–46. <https://doi.org/10.1111/j.1465-3362.2010.00279.x>
31. Driessen, M. (2001). The course of anxiety, depression and drinking behaviours after completed detoxification in alcoholics with and without comorbid anxiety and depressive disorders. Alcohol and Alcoholism, 36(3), 249–255. <https://doi.org/10.1093/alcalc/36.3.249>
32. Eslami, A. A., Norozi, E., Hajihosseini, M., Ramazani, A. A., & Miri, M. R. (2018). Social cognitive theory as a theoretical framework to predict sustained abstinence 6 months after substance use treatment. Journal of Substance Use, 23(3), 300–306. <https://doi.org/10.1080/14659891.2017.1394382>
33. Flora, K. (2018). A review of the factors affecting the course and outcome of the treatment of substance use disorders. Journal of Substance Use, 24(2), 120–124. <https://doi.org/10.1080/14659891.2018.1549598>
34. Garland, E. L., Franken, I. H. A., & Howard, M. O. (2012). Cue-elicited heart rate variability and attentional bias predict alcohol relapse following treatment. Psychopharmacology, 222(1), 17–26. <https://doi.org/10.1007/s00213-011-2618-4>
35. Glass, J. E., McKay, J. R., Gustafson, D. H., Kornfield, R., Rathouz, P. J., McTavish, F. M., Atwood, A. K., Isham, A., Quanbeck, A., & Shah, D. (2017). Treatment seeking as a mechanism of change in a randomized controlled trial of a mobile health intervention to support recovery from alcohol use disorders. Journal of Substance Abuse Treatment, 77, 57–66. <https://doi.org/10.1016/j.jsat.2017.03.011>
36. Godley, M. D., Godley, S. H., Dennis, M. L., Funk, R. R., & Passetti, L. L. (2006). The effect of assertive continuing care on continuing care linkage, adherence and abstinence following residential treatment for adolescents with substance use disorders. Addiction, 102(1), 81–93. <https://doi.org/10.1111/j.1360-0443.2006.01648.x>
37. Goldstein, R. B., Bigelow, C., McCusker, J., Lewis, B. F., Mundt, K. A., & Powers, S. I. (2001). Antisocial behavioral syndromes and return to drug use following residential relapse prevention/health education treatment. The American Journal of Drug and Alcohol Abuse, 27(3), 453–482. <https://doi.org/10.1081/ADA-100104512>
38. González-Roz, A., Castaño, Y., Krotter, A., Salazar-Cedillo, A., & Gervilla, E. (2024). Emotional dysregulation in relation to substance use and behavioral addictions: Findings from five separate meta-analyses. International Journal of Clinical and Health Psychology, 24(3), 100502. <https://doi.org/10.1016/j.ijchp.2024.100502>
39. Gossop, M., Marsden, J., Stewart, D., & Rolfe, A. (1999). Treatment retention and 1 year outcomes for residential programmes in England. Drug and Alcohol Dependence, 57(2), 89–98. <https://doi.org/10.1016/S0376-8716(99)00086-1>
40. Graham, L., Das, J., Vitorio, R., McDonald, C., Walker, R., Godfrey, A., Morris, R., & Stuart, S. (2023). Ocular microtremor: A structured review. Experimental Brain Research, 241(9), 2191–2203. <https://doi.org/10.1007/s00221-023-06691-w>
41. Gray, C., & Argaez, C. (2019). Residential treatment for substance use disorder: A review of clinical effectiveness. Canadian Agency for Drugs and Technologies in Health. <https://www.ncbi.nlm.nih.gov/books/NBK541232/>
42. Groh, D. R., Jason, L. A., Davis, M. I., Olson, B. D., & Ferrari, J. R. (2007). Friends, family, and alcohol abuse: An examination of general and alcohol-specific social support. American Journal on Addictions, 16(1), 49–55. <https://doi.org/10.1080/10550490601080084>
43. Groh, D., Jason, L. A., Ferrari, J., & Halpert, J. (2011). A longitudinal investigation of the predictability of the three-factor model of the Important People Inventory. The American Journal of Drug and Alcohol Abuse, 37(4), 259–263. <https://doi.org/10.3109/00952990.2011.591017>
44. Gupta, Y., Shanmugam, C., K, P., Mandal, S., Tandon, R., & Sharma, N. (2025). Pediatric keratoconus. Survey of Ophthalmology, 70(2), 296–330. <https://doi.org/10.1016/j.survophthal.2024.10.003>
45. Gurnani, B., & Kaur, K. (2021). Modified drug regimen for ophthalmic anesthesia during COVID-19 pandemic: Revisiting pharmacological concepts. Indian Journal of Pharmacology, 53(2), 178–179. <https://doi.org/10.4103/ijp.ijp_89_21>
46. Gustafson, D. H., McTavish, F. M., Chih, M.-Y., Atwood, A. K., Johnson, R. A., Boyle, M. G., Levy, M. S., Driscoll, H., Chisholm, S. M., Dillenburg, L., Isham, A., & Shah, D. (2014). A smartphone application to support recovery from alcoholism. JAMA Psychiatry, 71(5), 566. <https://doi.org/10.1001/jamapsychiatry.2013.4642>
47. Hajihosseini, M., Norozi, E., Azimi, T., & Ahmadinia, H. (2019). Comparison of artificial neural networks and logistic regression for determination of social-cognitive factors impacting drug abstinence. Journal of Substance Use, 25(3), 295–300. <https://doi.org/10.1080/14659891.2019.1690060>
48. Higgins, J., & Thomas, J. (2023). Cochrane handbook for systematic reviews of interventions. Cochrane Training. <https://training.cochrane.org/handbook/current>
49. Hser, Y. I., Joshi, V., Anglin, M. D., & Fletcher, B. (1999). Predicting posttreatment cocaine abstinence for first-time admissions and treatment repeaters. American Journal of Public Health, 89(5), 666–671. <https://doi.org/10.2105/AJPH.89.5.666>
50. Jainchill, N., Hawke, J., de Leon, G., & Yagelka, J. (2000). Adolescents in therapeutic communities: One year posttreatment outcomes. Journal of Psychoactive Drugs, 32(1).
51. Januszko, P., Gmaj, B., Piotrowski, T., Kopera, M., Klimkiewicz, A., Wnorowska, A., Wołyńczyk-Gmaj, D., Brower, K. J., Wojnar, M., & Jakubczyk, A. (2021). Delta resting-state functional connectivity in the cognitive control network as a prognostic factor for maintaining abstinence: An eLORETA preliminary study. Drug and Alcohol Dependence, 218, 108393. <https://doi.org/10.1016/j.drugalcdep.2020.108393>
52. Jayarajan, A. P., Sharma, A., Sharma, R., Nirankari, V. S., Narayana, S., & Christy, J. S. (2022). Congenital corneal anesthesia: A case series. Indian Journal of Ophthalmology, 70(7), 2588–2591. <https://doi.org/10.4103/ijo.IJO_3001_21>
53. Jean, Y. K., Kam, D., Gayer, S., Palte, H. D., & Stein, A. L. S. (2020). Regional anesthesia for pediatric ophthalmic surgery: A review of the literature. Anesthesia & Analgesia, 130(5), 1351–1363. <https://doi.org/10.1213/ANE.0000000000004012>
54. Johnston, A. L., Thevos, A. K., Randall, C. L., & Anton, R. F. (1991). Increased severity of alcohol withdrawal in in-patient alcoholics with a co-existing anxiety diagnosis. Addiction, 86(6), 719–725. <https://doi.org/10.1111/j.1360-0443.1991.tb03098.x>
55. Kabisa, E., Biracyaza, E., Habagusenga, J. d’Amour, & Umubyeyi, A. (2021). Determinants and prevalence of relapse among patients with substance use disorders: Case of Icyizere Psychotherapeutic Centre. Substance Abuse Treatment, Prevention, and Policy, 16(1), 1–12. <https://doi.org/10.1186/s13011-021-00347-0>
56. Keyser-Marcus, L., Alvanzo, A., Rieckmann, T., Thacker, L., Sepulveda, A., Forcehimes, A., Islam, L. Z., Leisey, M., Stitzer, M., & Svikis, D. S. (2015). Trauma, gender, and mental health symptoms in individuals with substance use disorders. Journal of Interpersonal Violence, 30(1), 3–24. <https://doi.org/10.1177/0886260514532523>
57. Killeen, T. K., Baker, N. L., Davis, L. L., Bowen, S., & Brady, K. T. (2023). Efficacy of mindfulness-based relapse prevention in a sample of veterans in a substance use disorder aftercare program: A randomized controlled trial. Journal of Substance Use and Addiction Treatment, 152, 209116. <https://doi.org/10.1016/j.josat.2023.209116>
58. Klein, A. A., & Slaymaker, V. J. (2011). 12-step involvement and treatment outcomes among young women with substance use disorders. Alcoholism Treatment Quarterly, 29(3), 204–218. <https://doi.org/10.1080/07347324.2011.586288>
59. Klein, A. A., Slaymaker, V. J., Dugosh, K. L., & McKay, J. R. (2012). Computerized continuing care support for alcohol and drug dependence: A preliminary analysis of usage and outcomes. Journal of Substance Abuse Treatment, 42(1), 25–34. <https://doi.org/10.1016/j.jsat.2011.07.002>
60. Kolla, B. P., Schneekloth, T., Mansukhani, M. P., Biernacka, J. M., Hall-Flavin, D., Karpyak, V., Geske, J., & Frye, M. A. (2015). The association between sleep disturbances and alcohol relapse: A 12-month observational cohort study. The American Journal on Addictions, 24(4), 362–367. <https://doi.org/10.1111/ajad.12199>
61. Kornfield, R., Toma, C. L., Shah, D. V., Moon, T. J., & Gustafson, D. H. (2018). What do you say before you relapse? How language use in a peer-to-peer online discussion forum predicts risky drinking among those in recovery. Health Communication, 33(9), 1184–1193. <https://doi.org/10.1080/10410236.2017.1350906>
62. Laffaye, C., McKellar, J. D., Ilgen, M. A., & Moos, R. H. (2008). Predictors of 4-year outcome of community residential treatment for patients with substance use disorders. Addiction, 103(4), 671–680. <https://doi.org/10.1111/j.1360-0443.2008.02147.x>
63. Lenzi, L., Ibrahim, A., Brough, D., & Thompson, A. (2025). Digital interventions for supporting alcohol abstinence in aftercare: A systematic review. Internet Interventions, 40, 100832. https://doi.org/10.1016/j.invent.2025.100832
64. Li, M. Y., Fei, Y. D., Zhang, X. X., Chen, T. W., Li, J., Sun, X. L., & Wang, Z. Y. (2024). Application of propofol-remifentanil intravenous general anesthesia combined with regional block in pediatric ophthalmic surgery. BMC Anesthesiology, 24(1), 147. <https://doi.org/10.1186/s12871-024-02531-8>
65. Lin, L., & Chu, H. (2017). Quantifying publication bias in meta-analysis. Biometrics, 74(3), 785–794. <https://doi.org/10.1111/biom.12817>
66. Ludwig, F., Tadayon-Manssuri, E., Strik, W., & Moggi, F. (2013). Self-efficacy as a predictor of outcome after residential treatment programs for alcohol dependence: Simply ask the patient one question! Alcoholism: Clinical and Experimental Research, 37(4), 663–667. <https://doi.org/10.1111/acer.12007>
67. Majer, J. M., Beasley, C., Stecker, E., Bobak, T. J., Norris, J., Nguyen, H. M., Ogata, M., Siegel, J., Isler, B., Wiedbusch, E., & Jason, L. A. (2018). Oxford House residents’ attitudes toward medication assisted treatment use in fellow residents. Community Mental Health Journal, 54(5), 571–577. <https://doi.org/10.1007/s10597-017-0218-4>
68. Mandell, W., Edelen, M. O., Wenzel, S. L., Dahl, J., & Ebener, P. (2008). Do dimensions of therapeutic community treatment predict retention and outcomes? Journal of Substance Abuse Treatment, 35(3), 223–231. <https://doi.org/10.1016/j.jsat.2007.10.004>
69. Mantagos, I. S., Kleinman, M. E., Kieran, M. W., & Gordon, L. B. (2017). Ophthalmologic features of progeria. American Journal of Ophthalmology, 182, 126–132. <https://doi.org/10.1016/j.ajo.2017.07.020>
70. McHugh, T. A. (2019). Implications of inspired carbon dioxide during ophthalmic surgery performed using monitored anesthesia care. AANA Journal, 87(4), 285–290.
71. McKellar, J. D., Harris, A. H., & Moos, R. H. (2009). Patients’ abstinence status affects the benefits of 12-step self-help group participation on substance use disorder outcomes. Drug and Alcohol Dependence, 99(1–3), 115–122. <https://doi.org/10.1016/j.drugalcdep.2008.07.005>
72. McKetin, R., Kothe, A., Baker, A. L., Lee, N. K., Ross, J., & Lubman, D. I. (2018). Predicting abstinence from methamphetamine use after residential rehabilitation: Findings from the Methamphetamine Treatment Evaluation Study. Drug and Alcohol Review, 37(1), 70–78. <https://doi.org/10.1111/dar.12528>
73. Mericle, A. A., Mahoney, E., Korcha, R., Delucchi, K., & Polcin, D. L. (2019). Sober living house characteristics: A multilevel analyses of factors associated with improved outcomes. Journal of Substance Abuse Treatment, 98, 28–38. <https://doi.org/10.1016/j.jsat.2018.12.004>
74. Michaud-Germain, C., Tardif, P.-A., Nadeau, A., Gagnon, A.-P., & Mercier, É. (2020). Contribution of substance use in acute injuries with regards to the intent, nature and context of injury: A CHIRPP database study. Cureus. <https://doi.org/10.7759/cureus.10282>
75. Mikhail, M., Sabri, K., & Levin, A. V. (2017). Effect of anesthesia on intraocular pressure measurement in children. Survey of Ophthalmology, 62(5), 648–658. <https://doi.org/10.1016/j.survophthal.2017.04.003>
76. Miller, D., Lewis, S. R., Pritchard, M. W., Schofield-Robinson, O. J., Shelton, C. L., Alderson, P., & Smith, A. F. (2018). Intravenous versus inhalational maintenance of anaesthesia for postoperative cognitive outcomes in elderly people undergoing non-cardiac surgery. Cochrane Database of Systematic Reviews, 8(8), CD012317. <https://doi.org/10.1002/14651858.CD012317.pub2>
77. Miller, J. M., & Miller, H. V. (2011). Considering the effectiveness of drug treatment behind bars: Findings from the South Carolina RSAT evaluation. Justice Quarterly, 28(1), 70–86. <https://doi.org/10.1080/07418825.2010.506880>
78. Mo, C., & Deane, F. P. (2016). Reductions in craving and negative affect predict 3-month post-discharge alcohol use following residential treatment. International Journal of Mental Health and Addiction, 14(5), 761–774. <https://doi.org/10.1007/s11469-015-9626-2>
79. Mohankumar, A., & Rajan, M. (2023). Role of hyaluronidase as an adjuvant in local anesthesia for cataract surgery. Indian Journal of Ophthalmology, 71(7), 2649–2655. <https://doi.org/10.4103/ijo.IJO_2515_22>
80. Mulhauser, K., Weinstock, J., Van Patten, R., McGrath, A. B., Merz, Z. C., & White, C. N. (2019). Examining the stability of the UPPS-P and MCQ-27 during residential treatment for substance use disorder. Experimental and Clinical Psychopharmacology, 27(5), 474–481. <https://doi.org/10.1037/pha0000255>
81. Müller, A., Znoj, H., & Moggi, F. (2019). How are self-efficacy and motivation related to drinking five years after residential treatment: A longitudinal multicenter study. European Addiction Research, 25(5), 213–223. <https://doi.org/10.1159/000500520>
82. Mutter, R., Ali, M. M., Smith, K., & Strashny, A. (2015). Factors associated with substance use treatment completion in residential facilities. Drug and Alcohol Dependence, 154, 291–295. <https://doi.org/10.1016/j.drugalcdep.2015.07.004>
83. Mutschler, C., Junaid, S., Tellez, C., Franco, G., Gryspeerdt, C., & Bushe, J. (2021). Community-based residential treatment for alcohol and substance use problems: A realist review. Health & Social Care in the Community, 30(2). <https://doi.org/10.1111/hsc.13511>
84. National Institute of Health. (2021). Study quality assessment tools | National Heart, Lung, and Blood Institute (NHLBI). <https://www.nhlbi.nih.gov/health-topics/study-quality-assessment-tools>
85. Page, M. J., McKenzie, J. E., Bossuyt, P. M., Boutron, I., Hoffmann, T. C., Mulrow, C. D., Shamseer, L., Tetzlaff, J. M., Akl, E. A., Brennan, S. E., Chou, R., Glanville, J., Grimshaw, J. M., Hróbjartsson, A., Lalu, M. M., Li, T., Loder, E. W., Mayo-Wilson, E., McDonald, S., & McGuinness, L. A. (2021). The PRISMA 2020 statement: An updated guideline for reporting systematic reviews. BMJ, 372, n71. <https://doi.org/10.1136/bmj.n71>
86. Polcin, D. L., & Henderson, D. M. (2008). A clean and sober place to live: Philosophy, structure, and purported therapeutic factors in sober living houses. Journal of Psychoactive Drugs, 40(2), 153–159. <https://doi.org/10.1080/02791072.2008.10400625>
87. Polcin, D. L., Korcha, R. A., Bond, J., & Galloway, G. (2010). Sober living houses for alcohol and drug dependence: 18-month outcomes. Journal of Substance Abuse Treatment, 38(4), 356–365. <https://doi.org/10.1016/j.jsat.2010.02.003>
88. Polcin, D. L., & Korcha, R. (2017). Housing status, psychiatric symptoms, and substance abuse outcomes among sober living house residents over 18 months. Addictive Disorders & Their Treatment, 16(3), 138–150. <https://doi.org/10.1097/ADT.0000000000000105>
89. Ponzer, S., Johansson, S.-E., & Bergman, B. (2002). A four-year follow-up study of male alcoholics: Factors affecting the risk of readmission. Alcohol, 27(2), 83–88. <https://doi.org/10.1016/S0741-8329(02)00203-3>
90. Proctor, S. L., Wainwright, J. L., & Herschman, P. L. (2017). Importance of short-term continuing care plan adherence on long-term outcomes among patients discharged from residential substance use treatment. The American Journal of Drug and Alcohol Abuse, 43(6), 734–741. <https://doi.org/10.1080/00952990.2017.1329315>
91. Reif, S., George, P., Braude, L., Dougherty, R. H., Daniels, A. S., Ghose, S. S., & Delphin-Rittmon, M. E. (2014). Residential treatment for individuals with substance use disorders: Assessing the evidence. Psychiatric Services, 65(3), 301–312. <https://doi.org/10.1176/appi.ps.201300242>
92. Rifai, M. A., Moles, J. K., Lehman, L. P., & Van der Linden, B. J. (2006). Hepatitis C screening and treatment outcomes in patients with substance use/dependence disorders. Psychosomatics, 47(2).
93. Rollins, A. L., O’Neill, S. J., Davis, K. E., & Devitt, T. S. (2005). Substance abuse relapse and factors associated with relapse in an inner-city sample of patients with dual diagnoses. Psychiatric Services, 56, 1274–1281.
94. Sannibale, C., Hurkett, P., Van den Bossche, E., O’Connor, D., Zador, D., Capus, C., Gregory, K., & McKenzie, M. (2003). Aftercare attendance and post-treatment functioning of severely substance dependent residential treatment clients. Drug and Alcohol Review, 22(2), 181–190. <https://doi.org/10.1080/09595230100100624>
95. Savage, C., & McCabe, L. (1973). Residential psychedelic (LSD) therapy for the narcotic addict. Archives of General Psychiatry, 28(6), 808. <https://doi.org/10.1001/archpsyc.1973.01750360040005>
96. Shrier, L. A., Harris, S. K., Kurland, M., & Knight, J. R. (2003). Substance use problems and associated psychiatric symptoms among adolescents in primary care. Pediatrics, 111(6), e699–e705. <https://doi.org/10.1542/peds.111.6.e699>
97. Sinclair, D. L., Sussman, S., De Schryver, M., Samyn, C., Adams, S., Florence, M., Savahl, S., & Vanderplasschen, W. (2021). Substitute behaviors following residential substance use treatment in the Western Cape, South Africa. International Journal of Environmental Research and Public Health, 18(23), 12815. <https://doi.org/10.3390/ijerph182312815>
98. Statista Research Department. (2023, August 31). Topic: Global drug use. Statista. <https://www.statista.com/topics/7786/global-drug-use/#topicOverview>
99. Stellern, J., Xiao, K. B., Grennell, E., Sanches, M., Gowin, J. L., & Sloan, M. E. (2022). Emotion regulation in substance use disorders: A systematic review and meta-analysis. Addiction, 118(1). <https://doi.org/10.1111/add.16001>
100. Substance Abuse and Mental Health Services Administration (US). (2019, November). Early intervention, treatment, and management of substance use disorders. U.S. Department of Health and Human Services. <https://www.ncbi.nlm.nih.gov/books/NBK424859/>
101. Suter, M., Strik, W., & Moggi, F. (2011). Depressive symptoms as a predictor of alcohol relapse after residential treatment programs for alcohol use disorder. Journal of Substance Abuse Treatment, 41(3), 225–232. <https://doi.org/10.1016/j.jsat.2011.03.005>
102. United Nations Office on Drugs and Crime. (2023). World drug report 2023. <https://www.unodc.org/res/WDR-2023/WDR23_Exsum_fin_DP.pdf>
103. van Hagen, L. J., de Waal, M. M., Christ, C., Dekker, J. J. M., & Goudriaan, A. E. (2019). Patient characteristics predicting abstinence in substance use disorder patients with comorbid mental disorders. Journal of Dual Diagnosis, 15(4), 312–323. <https://doi.org/10.1080/15504263.2019.1667043>
104. Vanderplasschen, W., Kag, C., & Ekm, B. (2009). Determinants of relapse and re-admission among alcohol abusers after intensive residential treatment. Archives of Public Health, 67, 194–211.
105. Weiss, N. H., Kiefer, R., Goncharenko, S., Raudales, A. M., Forkus, S. R., Schick, M. R., & Contractor, A. A. (2021). Emotion regulation and substance use: A meta-analysis. Drug and Alcohol Dependence, 230, 109131. <https://doi.org/10.1016/j.drugalcdep.2021.109131>
106. Welsh, W. N. (2007). A multisite evaluation of prison-based therapeutic community drug treatment. Criminal Justice and Behavior, 34(11), 1481–1498. <https://doi.org/10.1177/0093854807307036>
